# Fc-engineered antibodies with immune effector functions completely abolished

**DOI:** 10.1371/journal.pone.0260954

**Published:** 2021-12-21

**Authors:** Ian Wilkinson, Stephen Anderson, Jeremy Fry, Louis Alex Julien, David Neville, Omar Qureshi, Gary Watts, Geoff Hale

**Affiliations:** 1 Absolute Antibody Ltd, Wilton, United Kingdom; 2 mAbsolve Limited, Oxford, United Kingdom; 3 ProImmune Limited, Oxford, United Kingdom; 4 Antibody Analytics Limited, Motherwell, Scotland; 5 Reading Scientific Services Limited, Reading, United Kingdom; 6 Celentyx Limited, Birmingham, United Kingdom; 7 Abzena Limited, Babraham, United Kingdom; King’s College London, UNITED KINGDOM

## Abstract

Elimination of the binding of immunoglobulin Fc to Fc gamma receptors (FcγR) is highly desirable for the avoidance of unwanted inflammatory responses to therapeutic antibodies and fusion proteins. Many different approaches have been described in the literature but none of them completely eliminates binding to all of the Fcγ receptors. Here we describe a set of novel variants having specific amino acid substitutions in the Fc region at L234 and L235 combined with the substitution G236R. They show no detectable binding to Fcγ receptors or to C1q, are inactive in functional cell-based assays and do not elicit inflammatory cytokine responses. Meanwhile, binding to FcRn, manufacturability, stability and potential for immunogenicity are unaffected. These variants have the potential to improve the safety and efficacy of therapeutic antibodies and Fc fusion proteins.

## Introduction

Pharmacologic properties of immunoglobulins depend very much on their Fc region. Interaction with C1q initiates activation of complement. Binding to various Fc receptors on leucocytes induces antibody-dependent cell-mediated cytotoxicity (ADCC) or antibody-dependent cell-mediated phagocytosis (ADCP). Binding to the FcRn receptor is responsible for the comparatively long half-life of IgG. For many years, scientists have investigated the particular residues involved in binding to these various ligands, with the aim of modifying the natural properties of antibodies—either to enhance (e.g. to improve killing of tumour cells, or to extend half-life) or to reduce (e.g. to avoid unwanted side effects) [1–4]. Many therapeutic IgG4 antibodies have been developed as it was once believed that this isotype was devoid of effector function. However, that is not the case, as was tragically shown by a disastrous trial of the IgG4 CD28 antibody TGN1412 [5]. Alterations to the Fc region are necessary if binding to Fcγ receptors is to be eliminated. The Fc region is glycosylated and the carbohydrate is easily eliminated by mutation of N297, resulting in a reduction in binding to Fc receptors and C1q [6]. At least three therapeutic antibodies using the N297A mutation entered clinical trials (e.g. atezolizumab NCT02409355, clazakizumab NCT04343989, otelixizumab NCT01123083). However, removal of carbohydrate is a drastic change which significantly destabilises the Fc structure, leading to potential problems with manufacturability, stability and pharmacokinetics [7, 8].

One of the most widely used IgG1 variants is L234A/L235A (LALA) [9]. These substitutions reduce binding to the IgG Fc receptors FcγRI, FcγRII and FcγRIII as well as to complement component C1q. Such antibodies are useful where binding and activation of Fc receptors is undesirable, for example when the product is being used as an antagonist of a cytokine or similar. Numerous therapeutic antibodies using the LALA mutations have entered clinical trials (e.g. bimagrumab NCT01925209, cemiplimab NCT02383212, galcanezumab NCT03559257, progolimab NCT03912389, risankizumab NCT02684370, spesolimab NCT03482635, teplizumab NCT00385697). However, it is now known that LALA variants still have substantial binding to Fc receptors and so numerous others have been developed in the quest to completely eliminate binding to the Fcγ receptors [10–18]. Throughout the subsequent discussion, unless otherwise stated, we will refer to variants of human IgG1.

L234F/L235E/P331S (FES) and L234F/L235Q/K322Q (FQQ) are among the candidates [10, 11]. At least three antibodies with FES substitutions have entered clinical trials (e.g. anifrolumab NCT02446899, durvalumab NCT02369874, olendalizumab NCT01883544). However, in our tests, both variants still show significant levels of binding as well as being active in cell-based assays for the activation of receptors involved in ADCC and ADCP. An alternative set of variants is based on the substitutions A330S/P331S [9]. Serine is naturally found at 330 and 331 in IgG4, but when incorporated into IgG2, results in reduced FcR binding. This was used in a number of antibodies (e.g.bococizumab NCT01968967, fremanezumab NCT02621931, ponezumab NCT00945672, tanezumab NCT00733902). However, concerns emerged over the heterogeneity of disulphide bonding in IgG2 and reduced binding to FcRn leading to a shorter half-life [13]. More recently, L234A/L235A/P329G (LALAPG) was said to ‘completely abolish’ immune effector functions [14]. Several antibodies with this set of mutations have been taken into clinical trials (e.g. cergutuzumab NCT02350673, cibisatamab NCT03866239, faricimab NCT03038880, RG7386 NCT02558140). Other variants reported to reduce binding to Fcγ receptors include: L234A/G237A, L234A/L235A/G237A, L234A/L235A/G237A/P238S/H268A/A330S/P330S, L234A/L235E, G236R/L328R, and L234A/L235A/K322A [15–18].

In the above reports, different methods were used to measure binding to Fc receptors and functional activity of the variant Fc regions, so it is impossible to objectively compare the different mutations. We have now prepared a collection of antibodies having identical Fab regions but altered Fc regions as previously described. We compare them with a large number of novel Fc variants and find that all of the previous Fc variants still have a measurable level of FcγR binding activity. In contrast, some of our novel variants show no detectable activity at all. We go on to show that the new variants are not significantly different from wild-type with regard to other important properties, including binding to FcRn, stability and manufacturability or risk of unwanted immunogenicity. These new variants may find many applications in research, diagnosis and therapy.

## Materials and methods

### Human tissues

Human material for cytokine release studies was obtained by Celentyx from participants with written consent and approval from London-South East Research Ethics Committee REC reference 16/LO/0601. Cell samples for in vitro immunogenicity studies were obtained by ProImmune from NHS Blood & Transplant donors with written consent. They were processed and stored under Human Tissue Authority Licence 1258.

### Nomenclature

The EU numbering system [19] is used throughout this article. Amino acid alterations are described thus: XnnnY, where X is the single letter code for the residue in the native amino acid sequence, nnn is the EU index position and Y is the single letter code for the replacement amino acid residue. The symbol Δ refers to a residue which is deleted. Some variants are referred to in short form: LALA (L234A/L235A), LALAPG (L234A/L235A/P329G), FES (L234F/L235E/P331S), FQQ (L234F/L235Q/K322Q), aglycosyl (N297A),

### Antibody design, expression and purification

Codon-optimised synthetic genes encoding the wild-type human IgG1 heavy chain constant region or variants thereof were provided by Genewiz in pUC57-Kan vectors. The purified DNA was digested with NheI/NotI restriction enzymes and the gene extracted for ligation into a pUV mammalian expression vector. Similarly, a codon-optimised synthetic gene for human kappa light chain constant region was ligated into a pUV vector. Synthetic genes encoding the VH and VL variable regions of anti-CD20 rituximab, anti-CD3 muromonab or anti-CD52 alemtuzumab were provided by Genewiz with NheI and AvaI restriction sites at the 5’ and 3’ ends for cloning. VH genes were digested, excised and ligated into the wild-type human IgG1 heavy chain pUV vector. VL genes were digested, excised and ligated into the human kappa light chain pUV vector.

To create variants, synthetic genes containing variant Fc regions were synthesised with KasI and SacII restriction sites at the 5’ and 3’ ends and digested, excised and ligated into the existing vector encoding the immunoglobulin heavy chain of anti-CD20. Variants of the anti-CD3 heavy chain or anti-CD52 heavy chain were generated by digesting the appropriate VH variable regions with NheI and AvaI followed by excision and ligation into anti-CD20 variant heavy chain vectors similarly digested with NheI and AvaI to remove the canti-CD20 VH sequence for replacement with anti-CD3 or anti-CD52.

Intact antibodies were produced by transient transfection of HEK293 cells with a mixture of the appropriate heavy chain expression vector with the corresponding light chain expression vector encoding the cognate light chain. HEK293 cells were grown in suspension in serum-free culture medium (ThermoFisher Cat. No. 12338026) and transfected using linear polyethyleneimine. The cells were cultured for 6 days at 37°C with shaking at 140 rpm in 5% CO_2_. Culture supernatants were analysed by non-reducing SDS-polyacrylamide gel electrophoresis (SDS-PAGE) followed by staining with Coomassie Blue. All samples gave a single band corresponding to a molecular weight of approximately 150 kDa. Antibodies were purified by affinity chromatography on Protein A (MabSelect SuRe, GE Life Sciences). Eluted antibodies were buffer exchanged into phosphate buffered saline (PBS) pH 7.2 and concentrated as required. The protein concentration was measured by absorbance at 280 nm using a Nanodrop ND-1000 spectrophotometer and adjusted to 1 mg/mL by dilution with PBS. The binding of CD3 and CD52 antibodies to recombinant antigens was verified by ELISA. Several separate transfection experiments were carried out. A selected list of samples is shown in Table 2.

### Binding to immobilised Fc receptors

Binding analysis was carried out by surface plasmon resonance at 25°C using a Biacore T200 instrument with HEPES-buffered saline containing EDTA and P20 detergent (HBS-EP+) running buffer. The instrument was controlled using BiaCore T200 software version 1.0 and data were analysed using BiEvaluation software version 4.1 and Microsoft Excel 2016 32-bit. Mouse monoclonal IgG1 anti-histidine antibody (Qiagen Cat. No. 34670) was immobilised to a CM5 chip by carbodiimide chemistry giving a response in the range 6455 to 7834 RU. A reference cell was activated and blocked with ethanolamine. Histidine-tagged recombinant Fc receptors (Table 1) were diluted to 5 μg/mL in HBS-EP+ and injected for 2 min at 10 μL/min.

**Table 1:** Recombinant histidine-tagged Fc receptors.

Receptor	Species	Supplier	Catalogue number
FcγRI (CD64)	human	Sino	10256-H08H
FcγRIIa (CD32a) R131 allele	human	Sino	10374-H08H
FcγRIIb (CD32b)	human	Sino	10259-H08H
FcγRIIIa (CD16a) F158 allele	human	Sino	10389-H08H
FcγRIIIa (CD16a) V158 allele	human	SanYou	PHA135
FcγRIIIb (CD16b) NA2 allele	human	Sino	11046-H08H
FcγRI (CD64)	mouse	Sino	50086-M08H
FcγRI (CD64)	rat	Sino	80016-R08H
FcγRI (CD64)	rabbit	Sino	65010-T08H
FcRn	human	Sino	CT009-H08H
FcRn	cynomolgus	Sino	CT031-C08H
FcRn	mouse	Sino	CT029-M08H

Test samples were diluted with running buffer to 100 μg/mL and injected for 1 min at 30 μL/min. Between each cycle, the chip was regenerated by injection of 10 mM glycine pH 3.0 for 0.5 min at 30 μL/min and recharged with a fresh injection of Fc receptor. At the beginning and end of each experiment, blank cycles of buffer alone were run. The mean SPR signal (relative to the reference cell) was measured approximately 5 seconds before the end of injection (response). The blank (buffer) response was subtracted to give a corrected response for each test sample. Statistical analysis was carried out using a two-tailed t-test, assuming unequal variances.

Binding to FcRn was measured by indirect capture of histidine-labelled FcRn of various species as described above, except that the running buffer was adjusted to pH 6.0 by titration with acetic acid. Binding to human FcRn was also measured by direct capture. Human FcRn was immobilised on a CM5 chip by EDC/NHS chemistry to give a response of 645.8 RU. Samples were diluted with HBS-EP+ pH 6.0, injected for 2 min at 30 μL/min and allowed to disassociate for 1 min. Between each cycle, the chip was regenerated by injection of HBS-EP+ pH 7.4 for 1 min. A baseline correction was calculated by interpolating the response of blank samples at the beginning and end of the run. To correct for any loss of binding capacity during the run, a normalization factor was calculated by interpolating the response of the wild-type reference sample of anti-CD20 antibody at the beginning and end of the run. The baseline-corrected responses were divided by the normalisation factor to give corrected and normalised responses which were expressed as a percentage of the response of the corresponding wild-type reference.

### Binding to immobilised antibodies

Human, cynomolgus monkey and mouse Fcγ receptors and FcRn were produced in HEK293 cells. FcRn was expressed as a heterodimer with β2 microglobulin. The extracellular domains had a C-terminal hexa-His-tag and were purified by immobilised Ni^2+-^affinity chromatography using a 1 mL HisTrap FF column (Cytiva Cat. No. 17531901) followed by size exclusion chromatography using a 120 mL HiLoad 16/600 Superdex 200 pg column (Cytvia Cat. No. 28989335). Samples were eluted in HBS-EP+ at pH 7.4 and the monomer peak were pooled and analysed by SDS-PAGE.

Binding analysis was carried out in HBS-EP+ pH 7.4 (for Fcγ receptors) or PBS-Tween pH 5.8 (for FcRn) at 25°C using a Biacore 8K. The instrument was controlled using Biacore 8K Control Software version 3.0.12.15655. Data were collected and analysed using Biacore Insight Evaluation Software version 3.0.12.15655. Streptavidin was immobilised on flow cells 1 and 2 of a Series S CM5 Sensor Chip by standard amine coupling chemistry. Biotin-labelled anti-human κ light chain (CaptureSelect, Life Technologies Cat. No. 7103302100) was injected until an acceptable level (798 to 1392 RU) was achieved. Test samples, either Remicade (rituximab, wild-type human IgG1) or variant 2–52, were diluted with running buffer to 5 μg/mL and injected for 90 s at 10 μL/min on flow cell 2. Human, cynomolgus monkey, and murine Fcγ receptors were diluted to an optimal concentration for maximal binding to wild-type human IgG1, ranging from 12.5 nM (human and cynomolgus FcγRI) to 18 μM (human FcγRIIb). Human, cynomolgus and mouse FcRn were diluted to 1,500 nM, 500 nM, and 250 nM, respectively. The binding of FcRn was assessed over a 6-point, 1 in 2-fold dilution series (except for the murine FcRn: 5-point dilution series), in duplicate. Association and dissociation were set at 50 s and 60 s. Between each cycle, the chip was regenerated by injection of 10 mM glycine HCl pH 1.5 and 10 mM NaOH for 1 min at 50 μL/min. At the beginning and end of each experiment, blank cycles of PBS-Tween (pH 5.8) alone were run. After reference and blank subtractions, affinity constants were calculated using a non-linear curve fit equilibrium model (human and cynomolgus monkey FcRn) or a Langmuir 1:1 kinetic model (mouse FcRn).

### Fc effector cell bioassays

Antibodies were assessed for their ability to engage in antibody-dependent cell-mediated phagocytosis (ADCP) or antibody-dependent cell-mediated cytotoxicity (ADCC) using Promega Fc effector bioassay systems. The assay kit contained CD20+ Raji target cells (Promega cat. no. G7016) and engineered Jurkat effector cells which stably express the desired Fc receptor and an NFAT response element to drive expression of firefly luciferase. Experiments were carried out according to the manufacturer’s instructions using effector cells as listed in Table 2

**Table 2:** Effector cells.

Receptor	Species	Supplier	Catalogue number
FcγRI (CD64)	human	Promega	CS1781C01
FcγRIIa (CD32a) H131 allele	human	Promega	G988A
FcγRIIb (CD32b)	human	Promega	CS1781E01
FcγRIIIa (CD16a) V158 allele	human	Promega	G701A

Target cells, effector cells and sample dilutions were all prepared in RPMI1640 culture medium containing 4% low IgG bovine serum (assay buffer). 75 μL of assay buffer was added to the edge wells of a white, flat-bottomed microplate and 25 μL of target cell suspension was added to the central wells. Sample dilutions were prepared in an off-line plate; 25 μL was transferred to the central wells of the assay plate and mixed on a plate shaker. 25 μL of effector cell suspension was added and the plate was incubated at 37°C in 5% CO_2_ for 6 hours, then equilibrated at room temperature for 15 min. 75 μL of luciferase assay substrate was added and luminescence was measured using a Glomax 96 luminometer (Promega).

### Binding to C1q

Antibody binding to human C1q was measured by ELISA. Human C1q (Sigma Cat. No. 20476) was coupled to horse-radish peroxidase (HRP) using Lightning-Link^®^ HRP Conjugation Kit (Innova Biosciences Cat No 701–0003). ELISA was carried out with shaking at room temperature (18 to 22°C). Samples of purified antibodies comprising variant Fc regions were diluted to 10 μg/mL in PBS. Negative controls consisted of PBS alone. Replicates of 100 μL each were added to 96-well clear Maxisorp microplates (Sigma Cat. No. M9410-ICS) and incubated for 60 min. The plates were rinsed with PBS containing 0.05% (v/v) Tween 20 (wash buffer) and 200 μL PBS containing 1% casein (block buffer) was added. The plates were incubated for 60 min, then rinsed with wash buffer. 100 μL HRP-labelled C1q at 100 ng/mL in block buffer was added. The plates were incubated for 90 min then washed four times with wash buffer and twice with water. 100 μL of 3,3’,5,5’-tetramethylbenzidine liquid substrate, supersensitive (TMB) (Sigma Cat. No. T4444) was added and the plates were incubated for 10 min. 50 μL of 1M sulphuric acid was added to stop the reaction and the absorbance was read at 450 nm with 620 nm subtraction using a microplate spectrophotometer (Anthos Labtec HT). At least 6 replicates of each sample were tested in each experiment and the mean absorbance for each sample was compared with the mean absorbance obtained with buffer alone using a one-tailed t-test, assuming unequal variances and the resulting probability (P) values were calculated. Results were flagged if they were significantly higher (p ≤ 0.05) than the response for buffer alone.

### Cytokine release

Human peripheral blood mononuclear cells (PBMC) were isolated from healthy donors by centrifugation over Ficoll-Paque PLUS (GE Healthcare Cat. No. 11778538) and washed with RPMI medium containing 1% heat-inactivated foetal bovine serum, depleted of bovine IgG (FBS) and 1% penicillin/streptomycin (PS). The PBMC were resuspended at 2×10^6^ per mL in RPMI containing 10% FBS and 1% PS (cRPMI). Anti-CD3 antibodies at 1 mg/mL in PBS were diluted with cRPMI to give concentrations of 20, 2, or 0.2 or 0.02 μg/mL. A negative control consisted of PBS diluted five-fold with cRPMI. Each sample was tested in triplicate. 100 μL of test sample was mixed with 100 μL of cell suspension in a 96-well round-bottom microplate and cultured at 37°C and 5% CO_2_ for 24 h. The plate was centrifuged at 400 × g for 5 min and the culture supernatants were collected. Cytokines (GM-CSF, IFNγ, IL-2, IL-4, IL-10 and TNFα) were quantified by Luminex assay (Bio-Rad Cat. No. M50000007A) on a Bio-Plex^®^ 200 system with Bio-Plex Manager software according to manufacturer’s instructions. For each donor, the median value of the triplicate response was calculated. For each test sample, the median responses for the five donors were compared with the median responses for the negative control using a single-tailed, paired Student’s t-test to determine the significance of any differences. The distribution of each set of responses for the five donors was tested using the Shapiro-Wilk test and in the majority of cases gave a p-value >0.05 indicating the assumption that the data are normally distributed can be retained. The exceptions were limited to samples with responses very close to baseline. The complete dataset (i.e. 15 samples consisting of three replicates with each of five donors) was also analysed using the non-parametric single-tailed, paired Wilcoxon signed-rank test to confirm significant differences between test samples and the negative controls.

### Potential for binding to MHC Class II

The potential for variant Fc regions to give rise to peptides with the theoretical capacity to bind to human MHC Class II was assessed using the MHC-II Binding Prediction tool provided by the Immune Epitope Database (IEDB), available at http://tools.iedb.org/mhcii/. The prediction method was IEDB recommended version 2.22 [20]. This used a Consensus method, version 2.22 combining NN-align, SMM-align, CombLib and Sturniolo if any corresponding predictor was available for the molecule, otherwise NetMHCIIpan. The input consisted of a list of 16 sequences (wild-type and variants) corresponding to the Fc region of human IgG1, i.e. residues E216 to K448 inclusive. A reference set of 27 MHC Class II alleles [21] was selected with the default peptide epitope length of 15 amino acid residues. The output was sorted by rank score. A peptide was considered “at risk” of binding to MHC Class II if it gave a rank score of 10% or less for binding to any of the selected alleles. The total number of ‘at risk’ peptide/allele combinations was calculated for each variant.

### Potential for immunogenicity in vitro

The ProImmune ProMap^®^ T cell proliferation assay was used to assess the potential immunogenicity of peptides derived from variant Fc regions. 20-mer peptides were synthesized by Kendall Scientific Inc (Lincolnshire, IL, USA), purified by HPLC and analysed by HPLC and mass spectrometry to confirm purity (> 95%) and molecular weight (within 1Da of theoretical). The test samples were provided as freeze dried TFA salts and were dissolved in DMSO to give 1 mM. Two of the peptides were synthesized twice. The analyst was blinded to the peptide sequences and duplicates. PBMC were isolated from 20 healthy human donors and depleted of CD8+ T cells. Each sample was HLA-typed and stored in liquid nitrogen prior to use. They provided both T cells and antigen presenting cells (APC) in the assay. Cells were labelled with carboxyfluorescein succinimidyl ester (CFSE) prior to incubation with test peptides. Six replicates of each peptide were tested at a final assay concentration of 5μM. Controls included: (a) whole proteins Tuberculin Purified Protein Derivative (PPD), 5 μg/mL (b) Keyhole Limpet Hemocyanin (KLH), 0.25 mg/mL (c) ProMix^™^ CEFT Peptide Pool, 5 μM (d) a pool of peptides from KLH, 5 μM (e) culture medium alone. After 7 days of culture, cells were stained with anti-CD4 antibody, washed and fixed for analysis by flow cytometry using FlowJo Software (TreeStar Inc.). Proliferation was determined by measuring a decrease in CFSE intensity.

The number of CD4+ CFSEdim (i.e. proliferating) cells was determined as a proportion of the total CD4+ population to determine percentage stimulation. The background percentage stimulation of between 0.05% and 3.78% for the individual donors was subtracted from the percentage stimulation of the test samples and a one-way analysis of variance (ANOVA) was calculated to determine (to a significance level of p ≤ 0.05) whether the stimulation obtained from each peptide differed significantly from the unstimulated control. A sample was flagged ‘positive’ if all the following were true: (a) Percentage Stimulation ≥ 0.5% above Background, (b) Percentage Stimulation ≥ 2 × SEM above Background, (c) test sample significantly different from background p ≤ 0.05. Conventionally, a positive response in two or more independent donors is considered indicative of a potential T cell epitope.

### Aggregation by size exclusion chromatography

Size-exclusion chromatography (SEC) was carried out using an Agilent 1100 series HPLC with a Superdex 200 Increase 5/150 GL column equilibrated in PBS, pH 7.2 at a flow rate of 0.2 mL/min. Absorbance was measured at 280 nm and the areas of the peaks corresponding to IgG monomer and dimer were measured as a percentage of the total eluted protein. Samples were analysed shortly after preparation and again after incubation at 40°C for 7 days and 14 days.

### Thermal stability using fluorimetry and static light scattering

Antibodies were assessed using the Uncle^®^ Biostability Platform (Unchained Labs, Pleasanton CA, USA) in accordance with the manufacturer’s instructions. Samples were subjected to thermal ramping from 25°C to 95°C at 0.3°C per minute. Intrinsic protein fluorescence and static light scattering (266 nm) were measured as indicators of protein unfolding and aggregation. Each sample was tested at 1 mg/mL in PBS and a sample volume of 9 μL. The whole experiment was carried out three times to obtain triplicate independent temperature profiles. The melting temperature (Tm) was defined as the major inflection point on the fluorescence versus temperature plot, i.e. the temperature as which the rate of change of fluorescence reached a maximum. The software algorithm was unable to reliably determine a Tm from some of the fluorescence traces (even though the profiles were very similar to their replicates) and these were dropped from the analysis. The aggregation temperature (Tagg) was defined as the temperature at which the light scattering started to increase.

### Glycosylation

N-glycans were released with rapid PNGase F (NEB Cat. No. P07010) essentially according to the manufacturer’s instructions. The released glycans were fluorescently labelled by reductive amination with 2-aminobenzoic acid (2AA) (42.5 mg/ml) and sodium cyanoborohydride (0.33M), final concentrations, in a sodium acetate/boric acid/tetrahydrofuran buffer at 80°C for 1 hour. The labelled oligosaccharides were purified by HILIC using DPA-6S solid phase extraction cartridges [22]. The purified labelled oligosaccharides were analysed using an Agilent 1260 Quaternary HPLC system with fluorescence detection using a BEH Amide Column, 3.5 μm, 2.1 mm × 150 mm (Waters Cat. No. 186004861) at 30°C. The column was eluted at 0.15 mL/min with acetonitrile:0.525M ammonium acetate pH 3.75:water from 85:10:5 to 50:15:35. A dextran hydrolysate labelled with 2-aminobenzoic acid was used as an external calibrant to calculate glucose unit values for each peak.

### Enzyme digests

Test samples (0.5 mg/mL final concentration) at were incubated with proteases for 24 h at 37°C as shown in Table 3. The final volume of each digest was 10 μL.

**Table 3:** Enzyme digestion conditions.

Enzyme	Supplier	Cat. No.	Final concentration (μg/mL)	Buffer
trypsin	Sigma	T1426	50	PBS
neutrophil elastase	Merck	324681	25	PBS
plasmin	Sigma	P1867	75	PBS
MMP-3	Enzo	ALX-201-042-C005	50	PBS, 2.5 mM CaCl_2_
MMP-12	R&D Systems	917-MS	50	PBS, 2.5 mM CaCl_2_
cathepsin G	Enzo	BML-SE283-0100	50	PBS, 2.5 mM CaCl_2_
MMP-7	Enzo	ABML-SE181-0010	50	PBS, 2.5 mM CaCl_2_

Digestion was stopped by addition of 3.5 μL of E-PAGE loading buffer 1 and the samples were incubated at 70°C for 10 min. 10 μL (3.7 μg protein) was applied to an 8% E-PAGE 48 polyacrylamide gel (Invitrogen Cat. No. EP04808). SeeBlue Plus2 pre-stained molecular weight standards were applied to the reference lanes. The samples were electrophoresed for 25 min on an E-Base electrophoresis device (Invitrogen Cat. No. EB-M03). The gel was stained with 0.03% Coomassie Brilliant Blue R in 10% acetic acid/40% methanol and destained with 8% acetic acid. Images were captured using an E-gel camera (Invitrogen).

The experimental work described in this paper was carried out between Nov 2019 and Jun 2021.

## Results

### Design and initial screening of immunoglobulin variants

Previous work identified at least 39 different residues in human IgG1 which are relevant for binding to Fcγ receptors [4, 23–28]. Residues between 232 and 239 received particular attention and many of the variants in the clinic include alterations in this area [9–11, 14–18]. With advances in gene synthesis and rapid transient expression, it is now possible to explore a wider range of potential changes. For the initial screens we focused on the high affinity receptor FcγRI because this provided the most sensitive measure of any residual binding activity. Preliminary experiments showed that certain substitutions at 234 and 235 combined with G236K or G236E gave low levels of binding to FcγRI, comparable with LALAPG. When the same substitutions were combined with G236R, binding to FcγRI became undetectable (S7 Table). In subsequent experiments the permissible substitutions at positions 234 and 235 were systematically tested to find a variant that gave the lowest levels of binding to Fcγ receptors without compromising any desirable properties. We avoided residues with structural liabilities (Asn, Cys, Met, Pro) or those which increased the in-silico immunogenicity score (Phe, Trp, Tyr). A panel of culture supernatants from 165 variants was screened for binding to FcγRI S1 Table). From these, 63 variants giving the lowest responses were purified for analysis in more detail in parallel with variants representing previously described mutations. Results from three separate experiments are shown in Table 4.

**Table 4:** Binding responses of purified CD20 antibodies binding to human FcγRI.

Sample	Amino acid alterations, Sample description and [Literature Reference]	Response (RU)				P value	Compare with LALAPG
		Expt 1	Expt 2	Expt 3	Mean		
Buffer	Running buffer (Blank subtraction cycle)	0.0	0.0	0.0	0.0	0.017	Lower
10.75	L234A/L235A/P329G (LALAPG) [14]	80.8	43.3	nd	62.1	0.791	Equivalent
2–1	wild type reference	nd	2573.5	2600.3	2586.9	< 0.001	Higher
2–2	L234A/L235A/G236R	6.3	1.9	5.2	4.4	0.018	Lower
2–3	L234A/G236R	87.1	49.3	74.7	70.4	0.910	Equivalent
2–4	L234A/L235S/G236R	0.0	-1.2	2.4	0.4	0.016	Lower
2–5	L234A/L235T/G236R	14.6	11.2	17.3	14.3	0.023	Lower
2–6	L234D/L235H/G236R	4.3	3.8	7.7	5.2	0.018	Lower
2–7	L234D/L235K/G236R	-1.4	0.1	1.8	0.2	0.016	Lower
2–8	L234D/G236R	134.7	77.7	99.9	104.1	0.154	Equivalent
2–9	L234D/L235Q/G236R	5.7	5.9	7.1	6.2	0.020	Lower
2–10	L234D/L235S/G236R	4.0	2.7	3.6	3.4	0.019	Lower
2–11	L234D/L235T/G236R	3.0	4.3	5.5	4.3	0.019	Lower
2–12	L234E/L235D/G236R	15.1	9.4	12.3	12.3	0.022	Lower
2–13	L234E/L235H/G236R	4.6	4.5	6.2	5.1	0.019	Lower
2–14	L234E/L235I/G236R	11.6	8.1	11.7	10.4	0.022	Lower
2–15	L234E/G236R	232.6	143.2	179.3	185.0	0.035	Higher
2–16	L234E/L235V/G236R	4.8	5.5	7.4	5.9	0.020	Lower
2–17	L234G/L235H/G236R	1.8	4.2	24.4	10.1	0.008	Lower
2–18	L234G/L235Q/G236R	4.4	5.2	6.1	5.3	0.020	Lower
2–19	L234G/L235S/G236R	-0.1	2.7	3.1	1.9	0.017	Lower
2–20	L234H/L235I/G236R	13.1	10.9	22.8	15.6	0.017	Lower
2–21	L234H/L235S/G236R	0.7	3.4	5.9	3.3	0.017	Lower
2–22	L234K/L235Q/G236R	2.9	6.1	5.8	4.9	0.019	Lower
2–23	L234K/L235R/G236R	0.6	2.8	3.2	2.2	0.017	Lower
2–24	L234K/L235S/G236R	-1.7	1.1	4.1	1.2	0.015	Lower
2–25	L234K/L235T/G236R	-1.8	-1.0	1.1	-0.6	0.016	Lower
2–26	L234K/L235V/G236R	-1.7	0.4	3.1	0.6	0.016	Lower
2–27	L234Q/L235A/G236R	1.0	1.9	3.6	2.2	0.017	Lower
2–28	L234Q/L235D/G236R	4.3	2.7	4.4	3.8	0.019	Lower
2–29	L234Q/L235H/G236R	2.2	6.7	8.1	5.7	0.017	Lower
2–30	L234Q/G236R	41.9	27.0	49.0	39.3	0.064	Equivalent
2–31	L234Q/L235Q/G236R	0.3	3.0	34.9	12.7	0.019	Lower
2–32	L234Q/L235R/G236R	-0.2	2.6	9.4	3.9	0.013	Lower
2–33	L234Q/L235S/G236R	-1.8	1.6	3.8	1.2	0.015	Lower
2–34	L234Q/L235T/G236R	2.1	1.9	1.4	1.8	0.018	Lower
2–35	L234Q/L235V/G236R	0.5	2.5	4.5	2.5	0.017	Lower
2–36	L234R/L235D/G236R	0.4	0.9	4.2	1.8	0.017	Lower
2–37	L234R/L235E/G236R	0.9	1.8	4.6	2.4	0.017	Lower
2–38	L234R/L235H/G236R	-1.2	0.7	2.3	0.6	0.016	Lower
2–39	L234R/L235I/G236R	-0.8	-0.2	1.2	0.1	0.017	Lower
2–40	L234R/L235K/G236R	0.0	2.4	3.9	2.1	0.017	Lower
2–41	L234R/G236R	1.2	3.9	10.7	5.2	0.014	Lower
2–42	L234R/L235Q/G236R	-1.0	3.4	7.3	3.3	0.014	Lower
2–43	L234R/L235R/G236R	-0.4	1.8	pd	0.7	0.016	Lower
2–44	L234R/L235T/G236R	-0.8	3.9	25.1	9.4	0.008	Lower
2–45	L234S/L235D/G236R	4.3	7.2	20.5	10.6	0.010	Lower
2–46	L234S/L235E/G236R	3.7	5.3	6.9	5.3	0.019	Lower
2–47	L234S/L235G/G236R	2.6	1.5	3.7	2.6	0.018	Lower
2–48	L234S/L235H/G236R	0.4	1.1	3.9	1.8	0.017	Lower
2–49	L234S/L235I/G236R	3.9	4.2	6.4	4.8	0.019	Lower
2–50	L234S/G236R	33.7	21.6	30.9	28.7	0.034	Lower
2–51	L234S/L235R/G236R	-1.1	1.9	1.6	0.8	0.017	Lower
2–52	L234S/L235T/G236R	-0.5	0.3	1.9	0.6	0.017	Lower
2–53	L234S/L235V/G236R	0.7	2.1	4.3	2.4	0.017	Lower
2–54	L234T/L235A/G236R	4.3	4.8	7.2	5.4	0.019	Lower
2–55	L234T/L235D/G236R	6.0	28.9	62.3	32.4	0.145	Equivalent
2–56	L234T/L235H/G236R	1.4	7.4	60.9	23.2	0.123	Equivalent
2–57	L234T/L235I/G236R	7.2	17.3	24.2	16.2	0.014	Lower
2–58	L234T/L235K/G236R	1.1	2.4	4.5	2.7	0.017	Lower
2–59	L234T/G236R	156.0	105.3	139.7	133.6	0.029	Higher
2–60	L234T/L235Q/G236R	2.2	2.3	4.5	3.0	0.018	Lower
2–61	L234T/L235R/G236R	0.2	1.6	3.5	1.8	0.017	Lower
2–62	L234T/L235S/G236R	0.8	0.9	2.5	1.4	0.017	Lower
2–63	L234T/L235T/G236R	1.3	1.5	pd	1.4	0.018	Lower
2–64	L234T/L235V/G236R	2.8	3.6	5.4	3.9	0.018	Lower
2–65	L234A/L235A (LALA) [9]	781.7	581.7	675.1	679.5	0.008	Higher
2–66	L234A/L235A/P329G (LALAPG) [14]	81.8	51.3	72.8	68.6	1.000	Equivalent
2–67	N297Q (aglycosyl) [6]	744.6	491.6	578.1	604.8	0.017	Higher
2–68	G236R/L328R [17]	8.8	24.9	65.0	32.9	0.154	Equivalent
2–69	L234A/G237A [15]	255.2	175.7	222.2	217.7	0.014	Higher
2–71	L234A/L235E [18]	239.4	164.0	217.8	207.1	0.015	Higher
2–72	L235V/F243L/R292P/Y300L/P396L [29]	2874.8	2370.4	2507.9	2584.4	0.003	Higher
2–73	D265A/P329A [23]	1018.1	782.4	889.5	896.7	0.006	Higher
2–74	L234A/L235A/K322A [18]	772.0	581.6	674.9	676.2	0.007	Higher
2–75	L234F/L235E/P331S	595.7	453.7	547.8	532.4	0.006	Higher
2–76	L234F/L235Q/K322Q	951.8	730.2	823.2	835.1	0.006	Higher
2–77	L234A/L235A/G237A/P238S/H268A/A330S/P331S [16]	98.9	67.8	94.5	87.1	0.238	Equivalent
2–78	E233P/L234V/L235A/G236⊗/A327G/A330S/P331S [12]	43.7	28.2	40.2	37.4	0.055	Equivalent
2–79	L235A/G236R	41.7	29.8	49.6	40.3	0.069	Equivalent
2–80	L235S/G236R	27.1	34.6	69.6	43.8	0.202	Equivalent
2–81	G236R [17]	494.2	351.4	418.5	421.4	0.011	Higher
2–83	L235G/G236R [46]	173.5	125.3	166.3	155.0	0.013	Higher
10.75	L234A/L235A/P329G (LALAPG) [14]	67.8	45.7	65.9	59.8	0.488	Equivalent
Buffer	Running buffer	-0.9	-0.9	-0.6	-0.8	0.017	Lower

Results are flagged as being either significantly lower (p ≤ 0.05), equivalent (p > 0.05) or significantly higher (p ≤ 0.05) than the LALAPG reference antibody. nd = not done, pd = point dropped due to abnormal sensorgram.

The data were compared with those for a LALAPG reference antibody, chosen because it gave the lowest level of binding of the previously available variants. Fifty-six of the new variants gave significantly lower binding than LALAPG and many of them were indistinguishable from the buffer control. In contrast, all of the previously published variants (Samples 2–65, 2–67 to 2–78) and controls (Samples 2–79 to 2–83) gave higher or indistinguishable binding compared with LALAPG. Sample 2–72, which contains mutations designed to enhance binding to FcγRIII [29], gave binding to FcγRI which was comparable to the wild-type reference.

**Fig 1:**
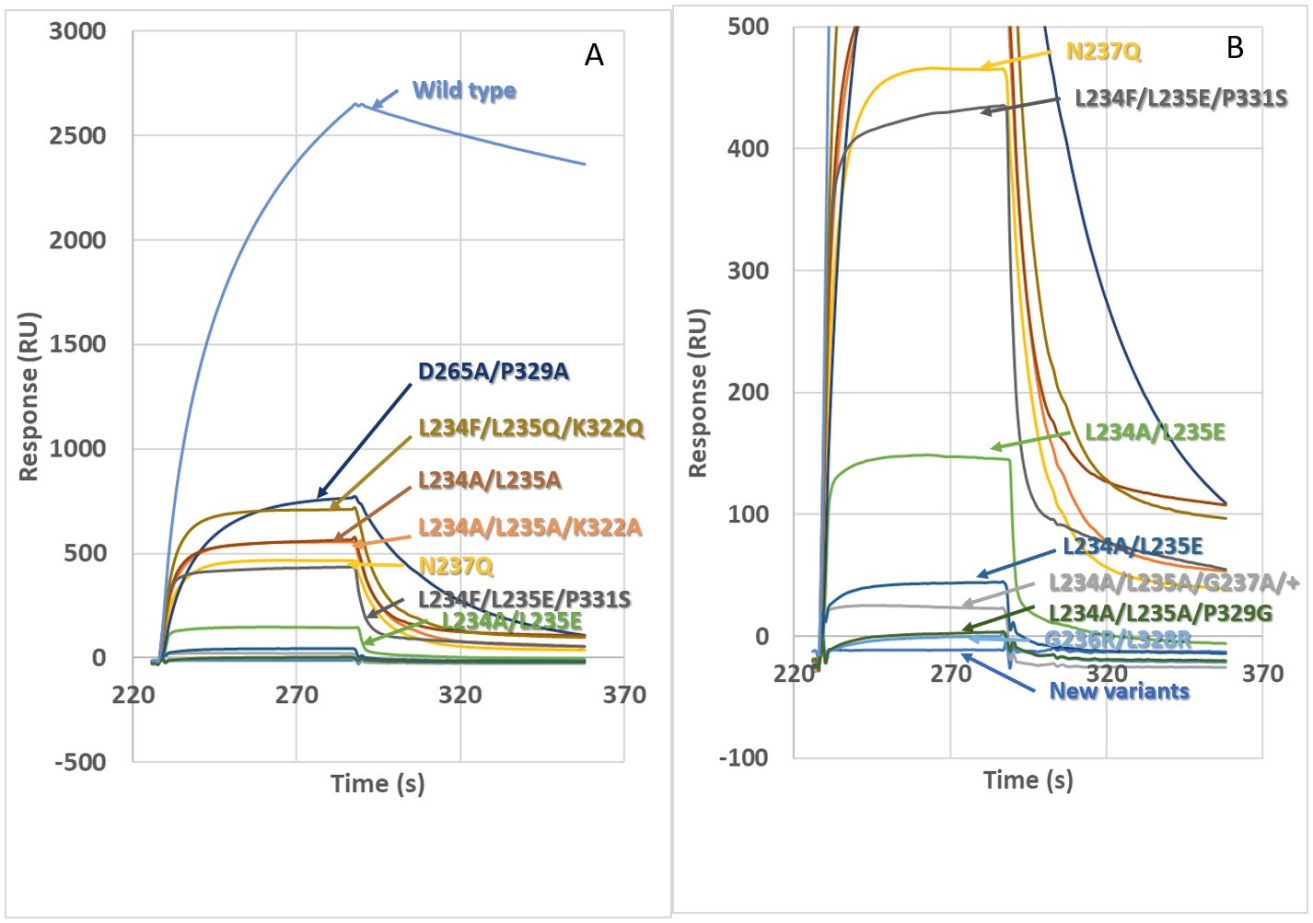
Sensorgrams showing binding of variant CD20 antibodies to FcγRI (CD64). Binding of a fixed concentration (100 μg/mL) of variant antibodies to immobilised receptor was measured by surface plasmon resonance. Sensorgrams were ‘double subtracted’, i.e. the response of a reference cell was subtracted from the response of a test cell to give the sample response and then the response of a buffer control was subtracted. For visualisation of high and low-level responses, the same data are shown at two different scales (Fig 1A and 1B). “New variants” include: L234G/L235S/G236R, L234S/L235T/G236R, L234S/L235V/G236R, L234T/L235Q/G236R and L234T/L235T/G236R, all of which gave baseline responses.

**Fig 2:**
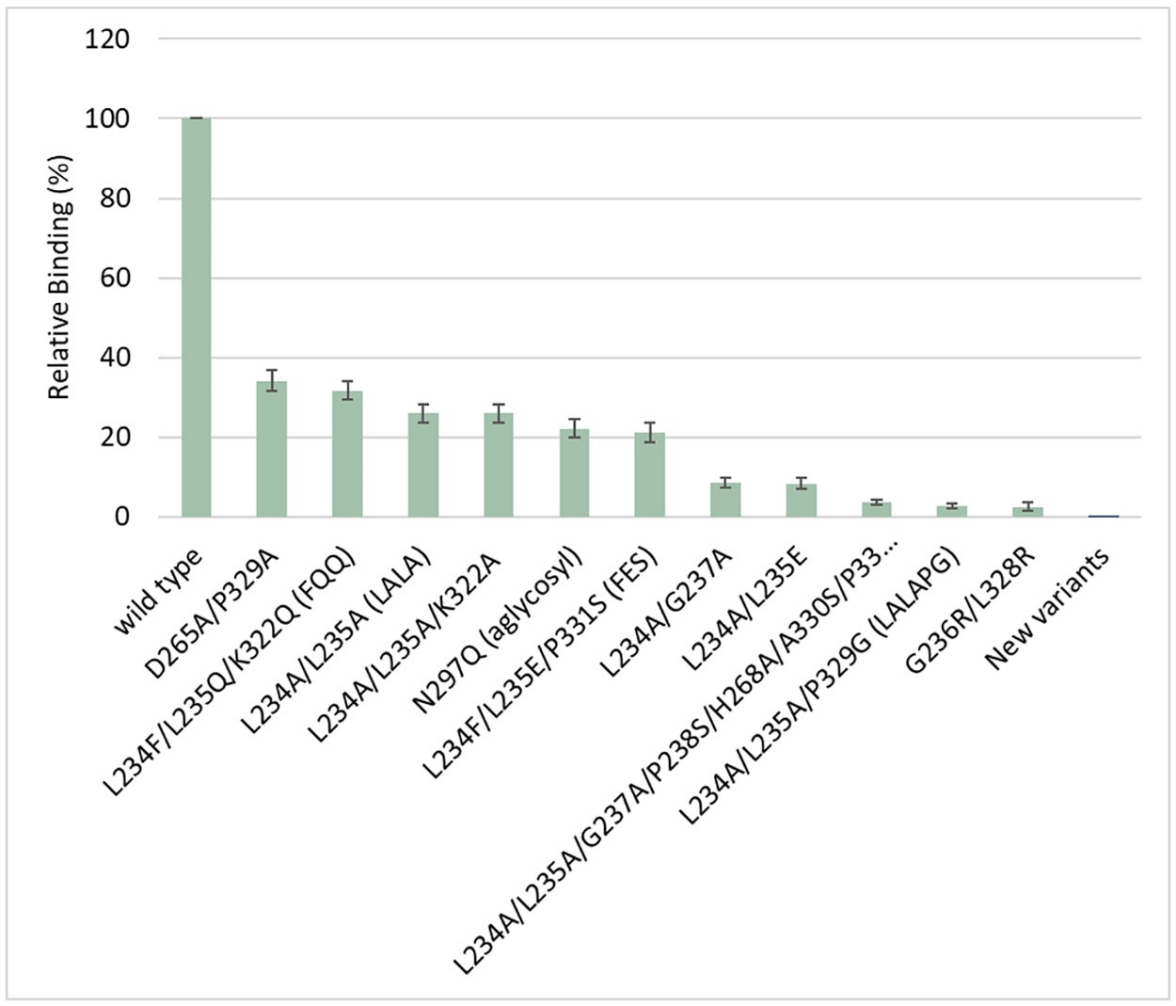
Binding of variant CD20 antibodies to FcγRI (CD64). The binding responses (measured at 283 s) for the selected samples shown in Fig 1 are expressed as a percentage of the binding of wild-type IgG1. Results are the mean ± standard deviation of two independent experiments (Expt 2 and Expt 3 in Table 4).

Selected sensorgrams are shown in Fig 1 to illustrate the range of responses seen with previously published variants. The mean responses for those variants are charted in Fig 2.

### Binding to Fc gamma receptors

Five of the new variants giving the lowest binding responses to FcγRI were selected for more detailed analysis on a range of receptors. They were re-expressed in combination with three different Fab regions, corresponding to rituximab (CD20), muromonab (CD3) and alemtuzumab (CD52). As controls, wild-type IgG1, LALA, LALAPG and aglycosyl variants were also expressed with each of the three variable regions. Each antibody was tested in duplicate for binding to each of the Fc receptors. The results are shown in Table 5. Significant binding of LALA, LALAPG and aglycosyl variants to human FcγRI was confirmed for all three antibody specificities. However, only aglycosyl antibodies gave significant binding to mouse and rat FcγRI. LALA variants showed significant binding to both alleles of FcγRIIIa and equivocal weak binding to FcγRIIa, FcγRIIb and FcγRIIIb. In contrast, all of the new variants gave very low binding responses with all of the receptors and were not significantly different from buffer alone.

**Table 5:** Binding responses of CD20, CD3 and CD52 antibodies binding to Fc gamma receptors.

			Receptor and allele							
Sample	Specificity	Amino acid alterations and sample description	human	human	human	human	human	human	mouse	rat
			FcγRI	FcγRIIa	FcγRIIb	FcγRIIIa	FcγRIIIa	FcγRIIIb	FcγRI	FcγRI
				R131		V158	F158	NA2		
			Mean response (RU)							
		PBS	1.6	0.5	0.9	1.5	0.5	0.8	-0.8	-1.1
2–1	CD20	wild type reference	2362.8	137.9	51.9	646.3	373.9	206.9	1935.8	789.0
2–19	CD20	L234G/L235S/G236R	-1.5	2.1	-0.1	1.0	1.0	-1.6	-2.1	-3.9
2–52	CD20	L234S/L235T/G236R	-1.3	2.6	0.5	-6.3	1.9	-3.0	-5.4	-2.4
2–53	CD20	L234S/L235V/G236R	-1.6	0.3	-0.8	0.8	0.0	0.6	0.1	-0.4
2–60	CD20	L234T/L235Q/G236R	-1.9	0.2	-1.1	-4.0	-0.5	-1.5	-4.1	-2.5
2–63	CD20	L234T/L235T/G236R	-2.7	1.9	0.6	-0.2	1.6	-0.1	-1.4	-0.8
2–65	CD20	L234A/L235A (LALA)	553.9	13.1	7.5	63.3	29.0	11.2	-12.2	-5.3
2–66	CD20	L234A/L235A/P329G (LALAPG)	48.1	3.0	4.1	1.5	4.7	5.1	2.4	0.7
2–67	CD20	N297Q (aglycosyl)	499.8	5.1	2.4	-0.9	2.4	2.7	59.5	559.9
3–1	CD3	wild type reference	2405.3	126.4	43.7	691.8	259.9	144.6	1995.2	789.6
3–19	CD3	L234G/L235S/G236R	-5.2	-4.8	-5.1	-14.4	-4.7	-6.4	-5.9	-4.2
3–52	CD3	L234S/L235T/G236R	-5.2	1.4	3.9	-11.3	3.3	4.7	-7.5	-5.1
3–53	CD3	L234S/L235V/G236R	-5.4	-3.4	-4.2	-12.7	-3.4	-4.8	-8.1	-4.8
3–60	CD3	L234T/L235Q/G236R	-3.2	-0.2	-2.8	-10.7	-0.9	-3.0	-5.8	-2.4
3–63	CD3	L234T/L235T/G236R	-3.1	-2.8	-3.3	-8.6	-2.0	-4.4	-5.5	-3.5
3–65	CD3	L234A/L235A (LALA)	534.5	5.8	-0.3	63.9	14.9	2.6	-3.3	-4.9
3–66	CD3	L234A/L235A/P329G (LALAPG)	39.4	-5.0	-5.6	-8.7	-3.8	not done	-6.2	-5.2
3–67	CD3	N297Q (aglycosyl)	502.0	1.7	-4.7	-13.3	-3.3	-2.8	43.9	478.5
4–1	CD52	wild type reference	2109.7	127.3	43.4	468.2	296.1	175.5	1621.4	593.6
4–19	CD52	L234G/L235S/G236R	-3.5	-3.5	-5.5	-2.4	-4.2	-4.8	-2.3	-1.7
4–52	CD52	L234S/L235T/G236R	-5.1	-3.1	-4.4	-5.6	-3.3	-4.3	-4.6	-2.9
4–53	CD52	L234S/L235V/G236R	-20.3	-4.2	2.4	-1.3	2.2	2.6	-2.5	-2.1
4–60	CD52	L234T/L235Q/G236R	-10.6	-2.2	1.3	-3.1	0.7	0.4	-1.4	0.5
4–63	CD52	L234T/L235T/G236R	-20.7	-8.7	-0.1	-2.0	-1.9	0.8	-3.9	-3.4
4–65	CD52	L234A/L235A (LALA)	451.5	5.8	2.3	63.2	17.0	5.9	1.8	-0.7
4–66	CD52	L234A/L235A/P329G (LALAPG)	33.7	1.2	2.9	-5.9	3.0	3.4	-2.1	-0.8
4–67	CD52	N297Q (aglycosyl)	430.6	3.1	2.9	-7.4	2.8	2.7	50.4	488.1

Test samples were analysed in duplicate. Between 11 and 14 samples of PBS were analysed and a cut-point was calculated equal to the mean + 1.645 × standard deviation 438 of these negative control samples. Test samples which gave a significantly higher response (p ≤ 0.05) are highlighted. The individual data, means and standard deviations are shown in S2 Table.

Additional experiments were carried out using an alternative Biacore assay format in which either wild-type IgG1 (Remicade) or variant 2–52 (L234S/L235T/G236R) was captured onto the Biacore chip and soluble Fc receptors were injected. An independently-made panel of receptors comprising: human FcγRI, FcγRIIa H131, FcγRIIa R131, FcγRIIIa V158, FcγRIIIa F158, FcγRIIIb; cynomolgus monkey FcγRI, FcγRIIa, FcγRIIb, FcγRIII; and mouse FcγRI, FcγRIIb, FcγRIII, FcγRIV, was tested to measure the binding of the 2–52 variant. All of the receptors gave positive binding responses of between 20 and 110 RU with the wild-type antibody whereas no significant binding (< 1 RU) was detected with the 2–52 variant.

### Fc effector cell activation assays

The ability of anti-CD20 antibodies to engage with cellular Fcγ receptors was measured using the Promega Fc effector cell bioassay reporter system to indicate the potential for antibody-dependent cell-mediated phagocytosis (ADCP) or antibody-dependent cell-mediated cytotoxicity (ADCC). Target cells were Raji, a CD20+ human B lymphocyte cell line and effector cells were Jurkat, a human T cell line with a luciferase gene under the control of a nuclear factor of activated T-cells (NFAT) promoter and transfected with the required human Fc receptor. In each assay, a dose response curve was established using wild-type IgG1 CD20 antibody. Samples of variant antibodies were tested at a final concentration (10 μg/mL) corresponding to maximal response of the control. The results are shown in Table 6. Consistent with the previous binding responses, LALA, LALAPG and aglycosyl variants all gave cellular responses via FcγRI significantly above the negative control and the LALA variant gave significantly elevated responses with all of the receptors. However, all of the new variants gave responses which were indistinguishable from buffer alone.

**Table 6:** Activity of CD20 antibodies in Fc receptor effector cell bioassays.

			Receptor and allele			
Sample	Specificity	Amino acid alterations and sample description	human	human	human	human
			FcγRI	FcγRIIa	FcγRIIb	FcγRIIIa
				H131		V158
			Mean normalised luminescence (% of wild-type reference)			
		assay buffer	2.0	2.2	1.8	2.8
2–1	CD20	wild type reference	100.0	100.0	100.0	100.0
2–19	CD20	L234G/L235S/G236R	2.0	2.0	1.7	2.5
2–52	CD20	L234S/L235T/G236R	1.7	2.2	1.7	2.2
2–53	CD20	L234S/L235V/G236R	1.9	2.9	1.7	2.1
2–60	CD20	L234T/L235Q/G236R	1.9	2.4	1.7	2.1
2–63	CD20	L234T/L235T/G236R	1.9	2.2	1.9	2.2
2–65	CD20	L234A/L235A	94.3	26.6	40.6	7.8
2–66	CD20	L234A/L235A/P329G (LALAPG reference)	4.9	2.3	1.7	2.1
2–67	CD20	N297Q (aglycosyl)	60.3	2.2	1.6	2.2

Samples which gave a significantly higher response (p<0.05) than the negative control (buffer alone) are highlighted.

### Binding to C1q

The binding of CD20, CD3 and CD52 antibodies to human C1q was measured by ELISA. Microplates were coated with test samples (or buffer alone) and incubated with peroxidase-labelled C1q. The results are shown in Table 7. Apart from very marginal responses from three of the CD52 samples, all of the variants gave binding responses which were indistinguishable from the buffer alone.

**Table 7:** Mean normalised absorbance of antibodies binding human C1q.

	Target antigen number of replcates		
Amino acid alterations and sample description	CD20	CD3	CD52
	12	12	12
	Mean normalised absorbance (% of wild-type reference)		
assay buffer	4.0	9.3	5.8
wild type reference	100.0	100.0	100.0
L234G/L235S/G236R	3.2	9.1	6.2
L234S/L235T/G236R	3.3	8.4	5.9
L234S/L235V/G236R	4.0	7.9	6.5
L234T/L235Q/G236R	4.2	8.3	6.0
L234T/L235T/G236R	3.7	8.6	6.5
L234A/L235A (LALA)	3.9	9.3	6.6
L234A/L235A/P329G (LALAPG reference)	4.5	8.5	6.5
N297Q (aglycosyl)	3.9	9.3	5.2

Each sample was tested in triplicate on four separate microplates. Samples which gave a significantly higher response (p ≤ 0.05) than the negative control (buffer alone) are highlighted. The mean and standard deviation of absorbance values for each microplate are shown in S3 Table.

### Cytokine release

**Fig 3:**
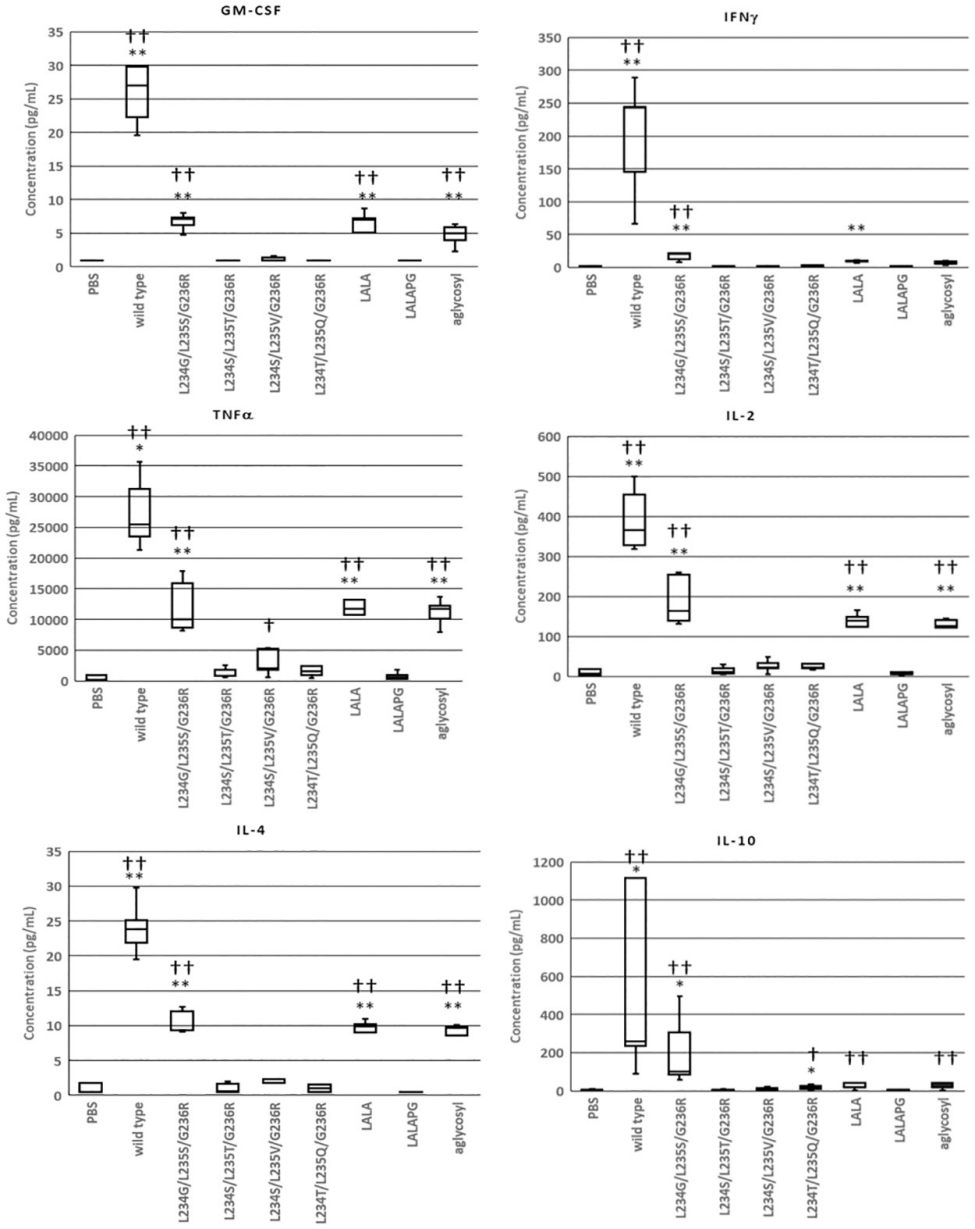
Release of cytokines from human PBMC exposed to CD3 antibodies. Cells were cultured for 24 h with variant antibodies at a final concentration of 10 μg/mL and the concentration of cytokines in the culture supernatant was measured by Luminex assay. The ‘box and whisker’ plots show the minimum, the first quartile, the median, the third quartile and the maximum values for the five donors. Test responses which were significantly different from the negative control by a paired t-test are shown thus: * p < 0.05, ** p < 0.01. Test responses which were significantly different from the negative control by a paired signed-rank test are shown thus: † p < 0.05, †† p < 0.01.

Samples of CD3 antibodies were tested for their ability to cause the release of inflammatory cytokines from human lymphocytes. Peripheral blood mononuclear cells (PBMC) from five donors were cultured with the test samples for 24 h and cytokines were measured in the supernatant by Luminex assay. The positive control (wild-type IgG1) was tested at a final concentration of 10, 1, 0.1 and 0.01 μg/mL and test samples at final concentration of 10 μg/mL. Release of GM-CSF, IFNγ, TFNα, IL-2, IL-4 and IL-10 from samples treated at 10 μg/mL are shown in Fig 3. The wild-type reference antibody gave positive responses at all concentrations and the LALA and aglycosyl variants also gave positive responses. However, variants L234S/L235T/G236R, L234S/L235V/G236R and L234A/L235A/P329G (LALAPG) gave no significant increase above the negative control for any of the cytokines. The same results were obtained whether the data were analysed by a parametric t-test or non-parametric signed rank test. Similar results were obtained from five independent donors tested at 0.1 μg/mL

### Binding to FcRn

The binding of CD20, CD3 and CD52 antibodies to human FcRn was measured by surface plasmon resonance with FcRn directly immobilised on a Biacore chip. Binding was measured at pH 6.0 and a sample concentration of 20 μg/mL. The results are shown in Table 8. The mean binding response of the three separate antibody specificities for the new variants was between 104.8% and 108.3% of the wild-type and the mean binding response of the controls was between 91.2% (aglycosyl) and 99.2% (LALA).

**Table 8:** Mean normalised binding of antibodies to human FcRn.

	Target antigen			Mean
Amino acid alterations and sample description	CD20	CD3	CD52	
	Mean normalised binding response (% of wild-type reference)			
wild type reference	100.0	100.0	100.0	100.0
L234G/L235S/G236R	101.6	102.4	117.7	107.2
L234S/L235T/G236R	101.1	104.0	115.8	106.9
L234S/L235V/G236R	104.9	100.8	119.1	108.3
L234T/L235Q/G236R	99.1	100.9	114.8	104.9
L234T/L235T/G236R	96.3	99.9	118.4	104.8
L234A/L235A (LALA)	96.2	96.8	104.8	99.2
L234A/L235A/P329G (LALAPG reference)	98.4	93.7	104.7	98.9
N297Q (aglycosyl)	86.7	85.1	103.3	91.7

Each sample was tested in duplicate. The individual normalised responses with mean and standard deviations are shown in S4 Table.

**Fig 4:**
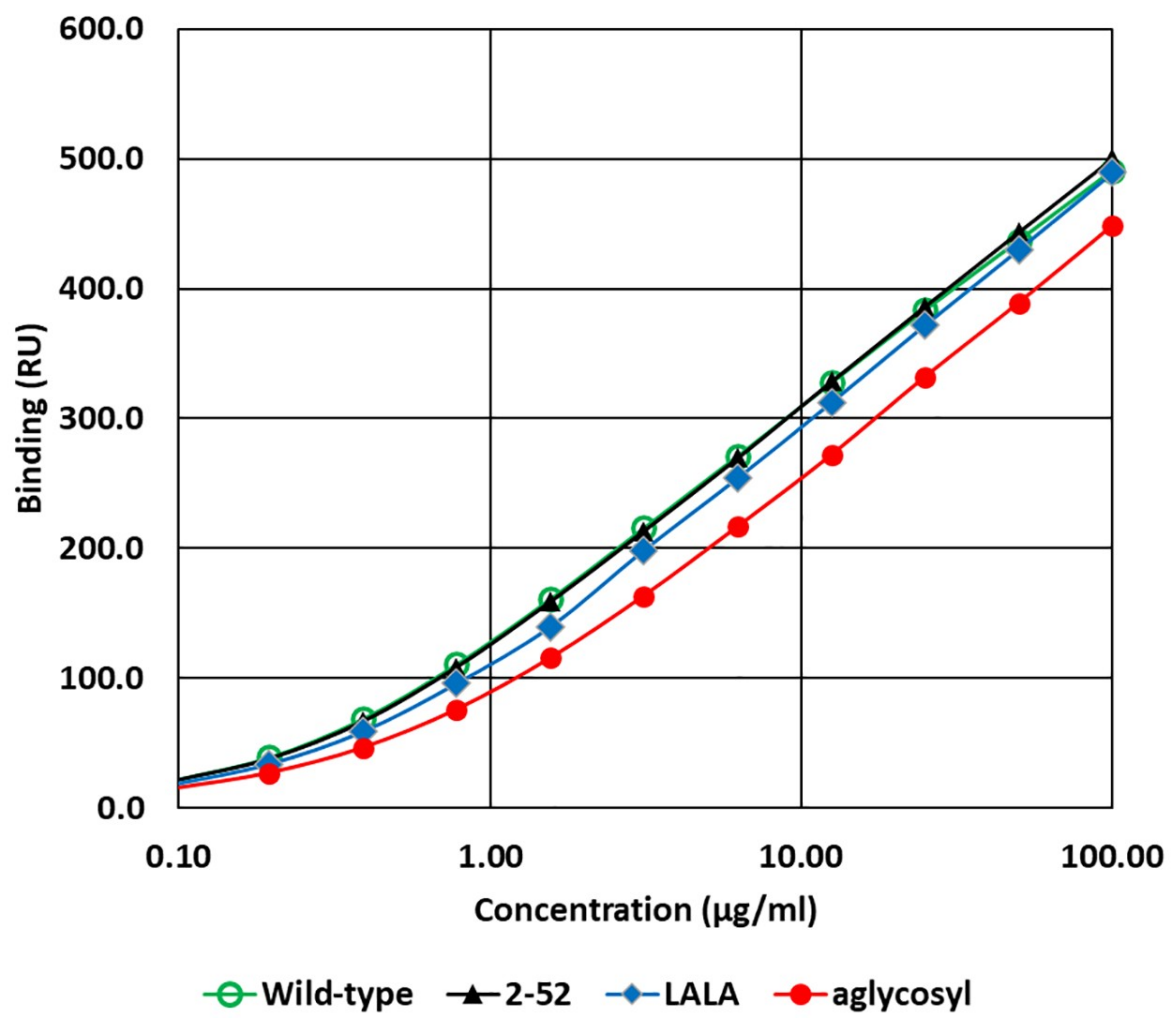
Dose response of binding of CD20 antibodies to human FcRn. FcRn was directly immobilised on a Biacore chip and various concentrations of CD20 antibodies (variants 2–52, 2–65 (LALA), 2–67 (aglycosyl) or wild-type) were injected at 25°C in HBS-EP+ at pH 6.0. Results are the mean of duplicates. Error bars are not shown as they were smaller than the symbols.

The dose response for selected samples is shown in Fig 4. The binding of wild-type and variant 2–52 (L234S/L235T/G236R) was selected as a representative sample of the new variants which performed particularly well in other tests. Its dose response curve was essentially indistinguishable from that of wild-type while the binding of 2–65 (LALA) and 2–67 (aglycosyl) were slightly lower.

**Fig 5:**
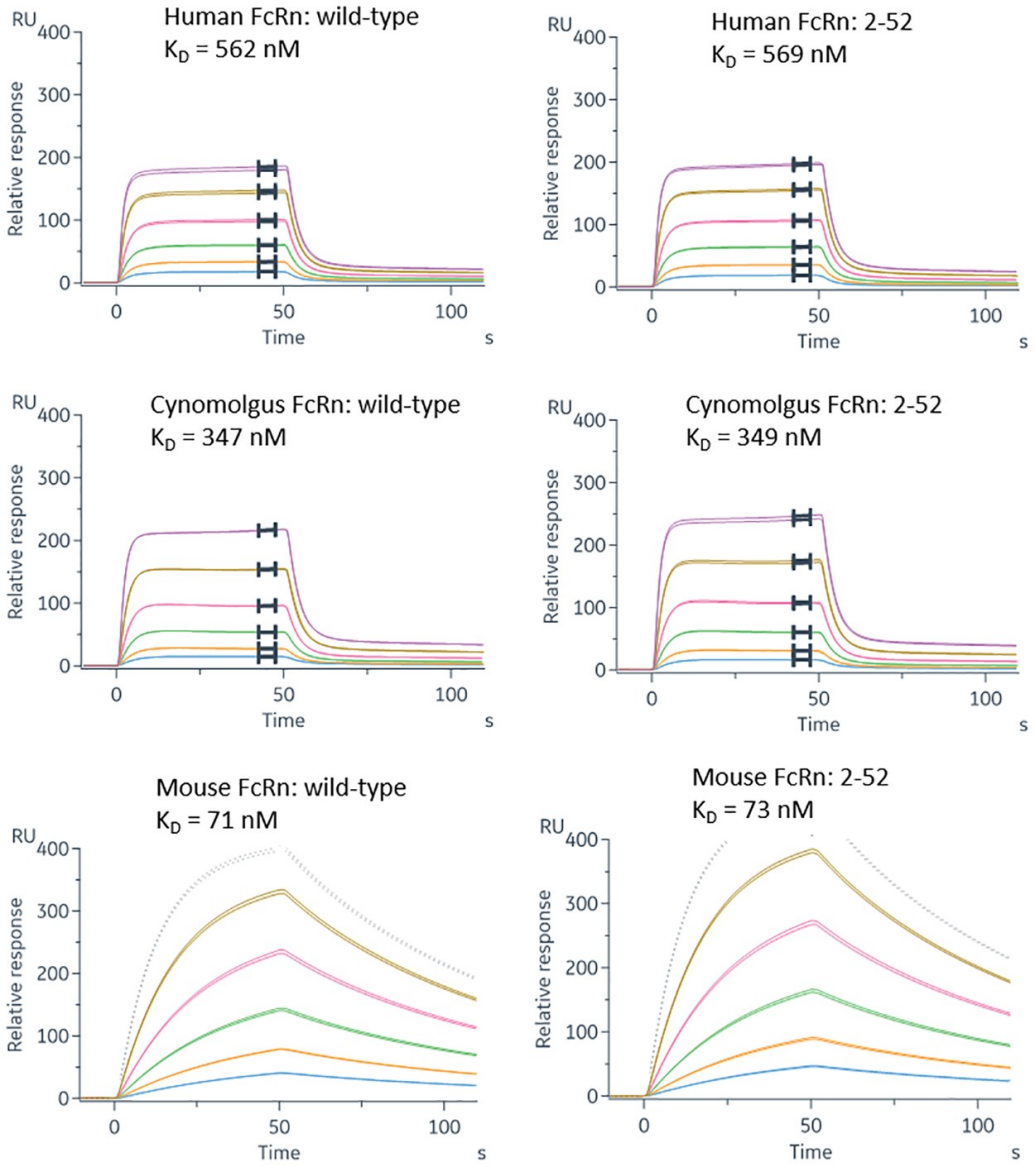
Binding of human, cynomolgus monkey or mouse FcRn to CD20 antibodies. Samples of wild type or variant 2–52 CD20 antibodies were captured by an anti-light chain antibody and various concentrations of soluble FcRn were injected at 25°C in PBS-T at pH 5.8. Dissociation constants (K_D_) were determined from equilibrium binding as a function of concentration (human and cynomolgus) or 1:1 kinetic fitting (mouse).

Additional experiments were carried out to compare the affinity of variant 2–52 (L234S/L235T/G236R) with wild-type IgG for binding to human, cynomolgus monkey and mouse FcRn using an alternative Biacore format with antibody immobilised on the chip and receptor in solution. The results are shown in Fig 5. The calculated dissociation constants (Kd) for the variant and wild-type antibody were essentially identical for each species.

### In silico assessment of potential binding to MHC Class II

The potential for variant Fc regions to give rise to peptides with the theoretical capacity to bind to major histocompatibility complex (MHC) Class II antigens was assessed using the IEDB prediction tool. A preliminary analysis of all possible substitutions (other than Cys) at positions 234 and 235 indicated that Ile, Leu, Met, Phe, Trp, Tyr or Val at 234 and Phe, Trp or Tyr at 235 were associated with an increased risk of binding to MHC Class II antigens. Subsequent analysis compared the Fc regions of selected variants. The results are shown in Table 9. Whilst several of the previously published variants had an excess of peptides with potential for binding to MHC Class II antigens (compared with wild-type IgG1), the new variants showed a marginal loss of potentially immunogenic peptides.

**Table 9:** Potential binding to MHC Class II.

Amino acid alterations and sample description	Binding score relative to wild-type
wild type IgG1	0
L234G/L235S/G236R	-3
L234S/L235T/G236R	-3
L234S/L235V/G236R	-3
L234T/L235Q/G236R	-3
L234T/L235T/G236R	-3
L234A/L235A (LALA)	2
L234A/L235A/P329G (LALAPG)	2
N297Q (aglycosyl)	-2
G236R/L328R	3
L234A/G237A	2
L234A/L235E	-3
D265A/P329A	19
L234A/L235A/K322A	20
L234F/L235E/P331S	-1
L234F/L235Q/K322Q	29
L234A/L235A/G237A/P238S/H268A/A330S/P331S	29
E233P/L234V/L235A/G236⊗/A327G/A330S/P331S	-10

### In vitro assessment of potential immunogenicity of peptides containing amino acid substitutions

In silico assessment did not take into account processing of peptides by antigen-presenting cells (APC). A T-cell proliferation assay was used to provide additional information. Peptides were synthesized which corresponded to residues 230 to 249 of wild-type IgG1 and various variants. PBMC from twenty donors were used as a source of APCs and CD4+ T cells and were cultured for 7 days with the test samples or controls. The analyst was blinded to the identity of the test samples and as a control, two of the peptides were synthesised twice and analysed separately (wild-type peptides 8 and 13 and L234S/L235T/G236R peptides 10 and 14). Proliferation of CD4+ cells was measured by flow cytometry. The results are summarized in Table 10. At least two independent donors should give a positive response for a peptide to be considered a potential T cell epitope. Control proteins and peptides gave the expected high level of stimulation. All the test samples gave very low responses, similar to buffer alone. Peptide 9 (G236R) gave four ‘positive’ responses and peptide 11 (L234S/L235V/G236R) gave two ‘positive’ responses. All of the ‘positive’ responses were weak with only low levels of percentage stimulation. Differences between the replicate samples (peptides 8 and 13 and peptides 10 and 14), which showed very low level responses by different donors reflected the inherent random noise level of the assay.

**Table 10:** Summary of responses for control and variant peptides in an in vitro immunogenicity assay.

Peptide number	Amino acid alterations and sample description	Responding Donors	Total responders	% Stimulation	ANOVA (p value)	Response Index
	**Controls**					
	PPD protein	1 to 20	20	3.4 to 61.5	< 0.001 to 0.002	33.5
	KLH protein	1 to 20	20	0.8 to 21.1	< 0.001 to 0.044	8.8
	CEFT peptide pool	1 to 20	20	3.6 to 44.7	< 0.001 to 0.002	19.4
	KLH peptide pool	1 to 7, 9 to 18, 20	18	0.7 to 24.8	< 0.001 to 0.035	6.7
	**Test samples**					
1	L234A/L235A/G237A		0	n/a	n/a	0
2	L234A/L235A (LALA)		0	n/a	n/a	0
3	L234A/L235E		0	n/a	n/a	0
4	L234A/G237A		0	n/a	n/a	0
5	L234F/L235E		0	n/a	n/a	0
6	L234F/L235Q		0	n/a	n/a	0
7	L234G/L235S/G236R		0	n/a	n/a	0
8	wild type IgG1 (sample 1)		0	n/a	n/a	0
9	G236R	1, 3, 18, 20	4	0.6 to 1.1	0.029 to 0.044	0.16
10	L234S/L235T/G236R (sample 1)	1	1	0.6	0.044	0.03
11	L234S/L235V/G236R	4, 10	2	0.6 to 0.7	0.039 to 0.040	0.07
12	L234T/L235Q/G236R		0	n/a	n/a	0
13	wild type IgG1 (sample 2)	10	1	1.1	0.049	0.06
14	L234S/L235T/G236R (sample 2)	4	1	0.7	0.022	0.04

### Aggregation by size exclusion chromatography

Samples of wild-type and variant CD20 antibodies were assessed by size-exclusion high pressure liquid chromatography (SEC-HPLC) to measure the proportion of monomer shortly after purification and again after incubation at 40°C for 7 days or 14 days. The results are shown in Table 11. Both the wild-type IgG1 and the variants showed a small decrease in monomer content at 7 days and a further small decrease at 14 days consistent with the appearance of dimers and/or higher order aggregates. Within the limits of this experiment there was no evidence for significant differences between the samples, except for a possible temperature-dependent increased aggregation of variant 2–33 (L234Q/L235S/G236R).

**Table 11:** Size exclusion chromatography of purified antibodies before and after incubation at 40°C.

Sample	Amino acid alterations and sample description	Fraction of IgG monomer (%)		
		Original	7 days at 40°C	14 days at 40°C
2–1	wild-type reference	97.7	96.5	93.9
2–19	L234G/L235S/G236R	98.1	97.5	93.9
2–33	L234Q/L235S/G236R	97.8	95.7	91.2
2–52	L234S/L235T/G236R	97.8	96.5	95.1
2–53	L234S/L235V/G236R	98.1	96.3	94.2
2–60	L234T/L235Q/G236R	98.2	94.6	94.1
2–63	L234T/L235T/G236R	97.7	95.1	95.1
2–65	L234A/L235A (LALA)	97.8	96.5	95.1
2–66	L234A/L235A/P329G (LALAPG)	97.7	96.2	94.2
2–67	N297Q (aglycosyl)	97.9	95.2	94.0

### Thermal stability by differential scanning fluorimetry and static light scattering

Antibodies were assessed using the Uncle^®^ Biostability Platform. Intrinsic protein fluorescence and static light scattering (266 nm) were measured as indicators of protein unfolding (Tm) and aggregation (Tagg) respectively. The results are shown in Table 12. CD52 antibodies generally had higher Tm and Tagg than CD20 and CD3 antibodies. The aglycosyl variants of CD20 and CD3 antibodies had significantly lower Tm and Tagg than their wild type equivalents and the variant L234Q/L235S/G236R showed a small amount of aggregation at a significantly lower temperature than wild type. All other variants had a Tm and Tagg either slightly higher than or not significantly different from the wild type.

**Table 12:** Melt temperatures (Tm) and onset of aggregation (Tagg) of purified antibodies.

Amino acid alterations and sample description	Target Antigen					
	CD20	CD3	CD52	CD20	CD3	CD52
	Mean Tm (°C)			Mean Tagg (°C)		
wild type reference	68.05	69.14	71.80	70.19	64.73	70.96
L234G/L235S/G236R	68.24^a^	70.53^b^	73.50	69.93	64.67	70.56
L234Q/L235S/G236R	69.39^a^	nd	nd	58.48	nd	nd
L234S/L235T/G236R	68.23^a^	72.07^a^	73.53	69.79	72.31	70.18
L234S/L235V/G236R	68.01	69.86^b^	73.88	68.41	64.20	69.96
L234T/L235Q/G236R	67.85	70.40^b^	73.25	69.03	66.11	69.91
L234T/L235T/G236R	68.28^a^	nd	nd	70.07	nd	nd
L234A/L235A (LALA)	68.85	69.45^b^	73.31	69.84	67.45	71.75
L234A/L235A/P329G (LALAPG)	67.27^a^	nd	nd	69.60	nd	nd
N297Q (aglycosyl)	59.14	60.00	73.23^a^	68.25	62.42	70.87

Results which were significantly (p ≤ 0.05) less than wild type are highlighted.

^a^Data available from only two replicates.

^b^Data available from only one replicate.

### Glycosylation

**Fig 6:**
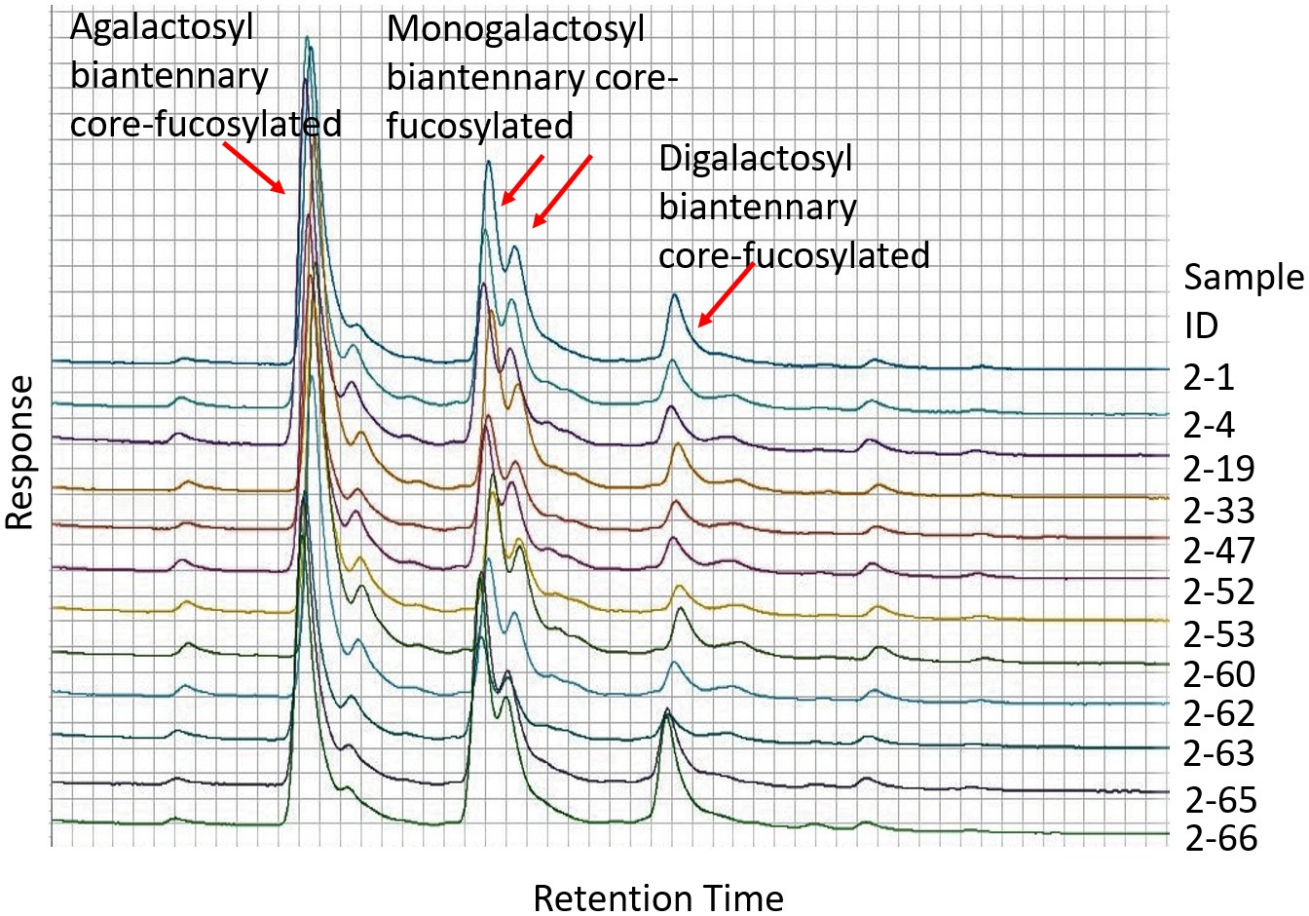
Overlay of representative chromatograms showing the carbohydrate composition of anti-CD20 antibodies derived from HEK293 cells. The major peaks are labelled with the corresponding structures determined by comparison with a control monoclonal antibody. Sample 2–1 is wild-type IgG, 2–65 is L234A/L235A (LALA) and 2–66 is L234A/L235A/P329G (LALAPG); other samples are new variants as listed in Table 4.

The N-linked carbohydrates of wild-type and variant CD20 antibodies were analysed by hydrophilic interaction liquid chromatography (HILIC). Results are shown in Fig 6.

All of the samples showed similar profiles with the biantennary structures typical of immunoglobulins. Slightly different profiles were seen for antibodies expressed in CHO cells but again the variant and wild-type antibodies were similar.

### Protease sensitivity

**Fig 7:**
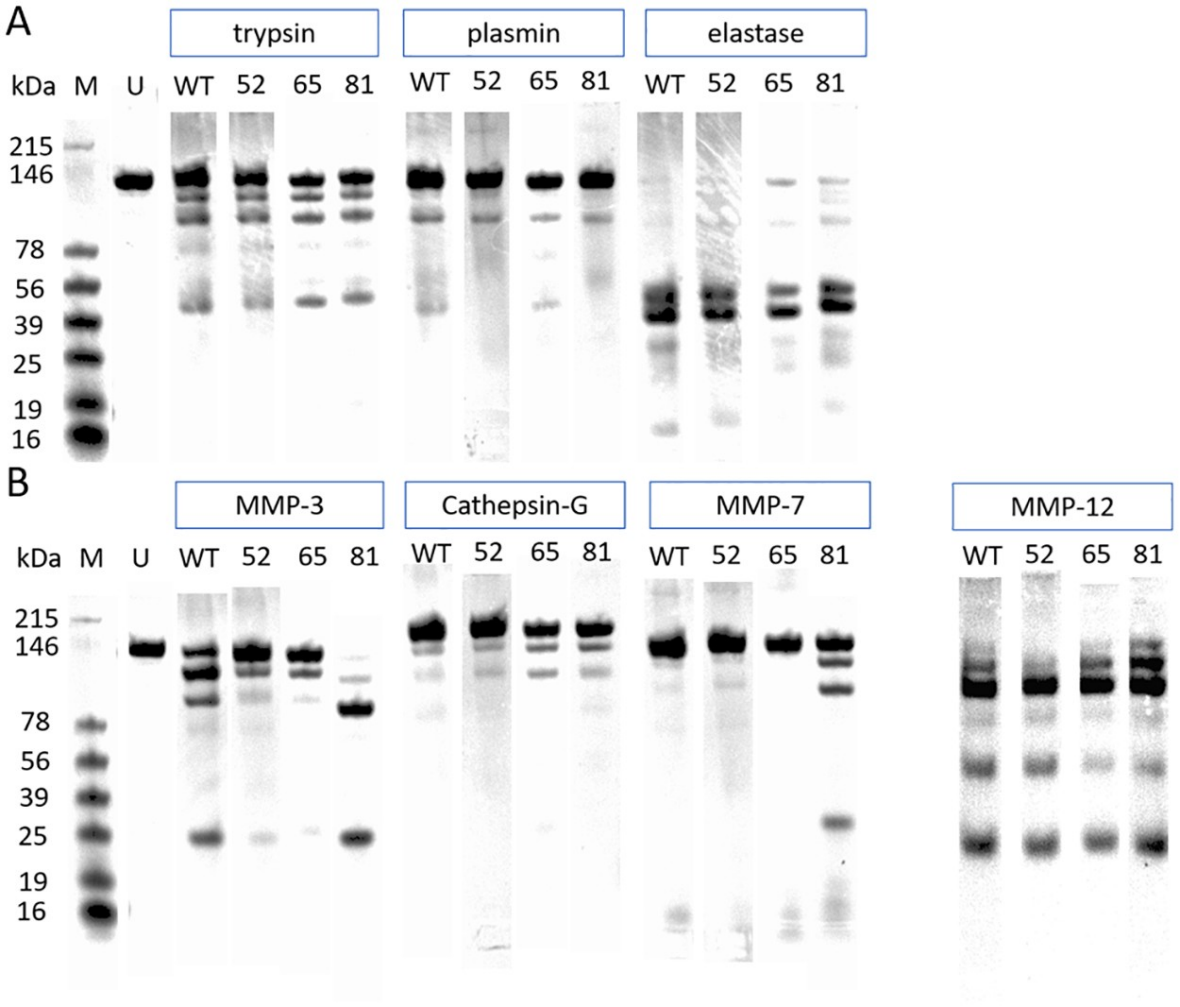
Digests of CD20 antibodies. M = marker, U = undigested sample, WT = wild-type IgG1, 52 = variant 2–52 (L234S/L235T/G236R), 65 = variant 2.65 (LALA), 81 = variant 2–81 (G236R). Individual lane images representative of six separate experiments were selected and re-ordered for purpose of illustration; the original gel images are available in S1 Fig. A: samples incubated with trypsin, pepsin and elastase were analysed on a single gel. B: samples incubated with MMP-3, Cathepsin-G and MMP-7 were analysed on one gel and samples incubated with MMP-12 were analysed on another.

The hinge region of immunoglobulins is sensitive to proteolysis. Matrix metalloproteases (MMPs) secreted by tumour cells can degrade therapeutic antibodies and possibly reduce their effectiveness [30, 31]. To test the impact of amino acid substitutions on protease sensitivity, selected antibodies were exposed to a range of proteolytic enzymes and analysed by gel electrophoresis. Results are shown in Fig 7. Digestion with trypsin, elastase, plasmin, MMP-12 and cathepsin G gave similar results with wild-type IgG and the variants. The substitution G236R did not change the pattern of cleavage by trypsin, which is already expected to cleave at K222. Variants 2–52 (L234S/L235T/G236R) and 2–65 (LALA) were cleaved less extensively by MMP-3 than wild-type. There was no significant cleavage of wild-type, L234S/L235T/G236R or LALA variants by MMP-7, but G236R was partially fragmented.

## Discussion

Since the first therapeutic monoclonal antibody, OKT3, entered the clinic in the early 1980s, side effects caused by unwanted inflammatory responses have been a complication of therapies which otherwise had great potential [32, 33]. Early research with recombinant antibodies suggested that the IgG4 isotype might be comparatively inert [34]. As a consequence, IgG4 antibodies have been selected for applications where inflammatory applications needed to be avoided [35, 36]. However, it was soon appreciated that the picture is not so simple due to the existence of multiple Fc gamma receptors, and different polymorphic forms, resulting in significant biologic activity for IgG4 in many situations [37, 38]. This became particularly evident with the disastrous results of administering the IgG4 anti-CD28 antibody TGN1412 [5, 39]. Attention shifted to the development of variant antibodies with Fc regions mutated to reduce binding to Fc gamma receptors and also to C1q [1–18]. Many variants have been described in the literature and several have reached the clinic, but their functional properties have not been systematically compared until now. To our surprise, we found that all of the previously described variants we tested still gave detectable binding at least to FcγRI, including those which had been described as “completely abolished” [14, 18], “silent” [16] or “no detectable binding” [15].

One of the key motifs involved in binding to Fcγ receptors is the region between residues 232 and 239 in the CH2 domain. We systematically explored amino acid substitutions at residues 234, 235 and 236 and found a novel set of substitutions where the binding to FcγRI was significantly less than the best of the previously available variants and in most instances below the limit of detection. Low levels of binding to Fcγ receptors shown by previous variants were associated with more substantial levels of activation in Fc effector cell bioassays as well as measurable cytokine release from human PBMC. However, some of the new variants were still effectively silent in all of these assays. In particular, variant 52 (L234S/L235T/G236R) consistently performed well in all of the binding and functional assays.

Of course, it is essential that mutations introduced into a therapeutic product do not have a deleterious effect on manufacturability, stability, pharmacokinetics or immunogenicity. The variants described here were produced by transient transfection of HEK293 cells. They all gave yields comparable with wild-type IgG1 (S5 Table). Similar results were obtained with variants produced by transfection of CHO cells (S6 Table). After purification by affinity chromatography on Protein A, the proportion of dimer, measured by SEC-HPLC, ranged between 1.8% and 2.3%. After 14 days at 40°C, there was still no significant difference between the dimer content of variants and wild-type IgG1 (range 4.9% to 6.1%) except for variant 2–33 (L234Q/L235S/G236R) which contained about 8.8% dimer. Stability was further examined by thermal scanning using intrinsic protein fluorescence to monitor unfolding (Tm) and light scattering to monitor aggregation (Tagg). Consistent with the SEC-HPLC results, variant 2–33 showed a slightly increased tendence to aggregate. Two aglycosyl antibodies also had a lower Tagg. The Tm and Tagg of all other variants were either greater than, or not significantly different from the corresponding wild-type IgGs. In particular, variant 52 (L234S/L235T/G236R), which had performed well in binding and functional assays, also demonstrated an excellent stability profile.

The long half-life of IgG in vivo is a consequence of rescue from catabolism by binding the neonatal receptor FcRn [40]. All variants, with the exception of aglycosyl, gave similar levels of binding to human FcRn and variant 52 (L234S/L235T/G236R) showed identical kinetics to wild-type IgG with mouse, monkey and human FcRn. Further experiments to determine pharmacokinetics in mice transgenic for human FcRn are underway and will be reported separately.

Unwanted immunogenicity is an important issue for therapeutic antibodies. Nevertheless, we are not aware of reports of clinically significant anti-drug antibodies directed against the human Fc region as a result of the numerous different mutations which have been introduced to modify Fc effector function, improve stability or to create bispecific antibodies. However, there remains a theoretic possibility that mutations might create novel T cell or B cell epitopes and result in the formation of anti-drug antibodies. It is notoriously difficult to predict immunogenicity, but various tools have been developed both for in silico prediction of peptides which might bind to MHC antigens and also for in vitro analysis of possible immune cell responses [41, 42]. So far as we can tell, using both in silico and in vitro methods, none of the new variants are associated with an increased risk of immunogenicity compared with wild-type IgG1.

Binding to Fc receptors on antigen presenting cells can lead to enhanced uptake and more effective presentation of foreign antigens [43–45]. Indeed this has been proposed as a strategy to create more effective vaccines. Therefore, it is plausible that elimination of binding to Fcγ receptors and particularly to FcγRI may reduce the potential immunogenicity of therapeutic antibodies and Fc fusion proteins. Hitherto, less attention has been given to reducing the binding to FcγRI and all of the variants used in the clinic have some residual level of binding activity (Fig 2).

There is a huge resurgence of interest in therapeutic applications of anti-CD3 antibodies, and particularly in the use of bispecific antibodies for retargeting T cells. With these products, complete suppression of Fc-mediated effector functions is required to minimize off-target toxicity and maximize therapeutic efficacy [46]. The same is true in many other situations, whether for treatment of cancer, autoimmune disease, infectious disease or others. Complete abrogation of Fc effector function offers the prospect of maintaining all the other useful properties of the Fc region (long half-life etc), and at the same time improving the safety and efficacy of antibodies and fusion proteins.

## Supporting information

S1 Fig Raw gel images used for preparation of Fig 7. (PDF)

S1 Table Initial screen of variant antibodies binding to human FcγRI. (PDF)

S2 Table Individual results for Table 5: Binding responses of CD20, CD3 and CD52 antibodies binding to Fc gamma receptors. (PDF)

S3 Table Mean and SD of absolute absorbance responses for Table 7: Mean normalised absorbance of antibodies binding human C1q. (PDF)

S4 Table Individual results for Table 8: Mean normalised binding of antibodies to FcRn. (PDF)

S5 Table Yield and purity of variant antibodies produced from HEK cells. (PDF)

S6 Table Yield and purity of variant antibodies produced from CHO cells. (PDF)

S7 Table Preliminary screen of variant antibodies binding to human FcγRI. (PDF)
