# Supplementary material for: Fc-engineered antibodies with immune effector functions completely abolished
Source: PLoS One. 2021 Dec 21;16(12):e0260954. doi: 10.1371/journal.pone.0260954 (PMC8691596; doi:10.1371/journal.pone.0260954)
Supplement: S1 Fig — (PDF) [file pone.0260954.s001.pdf]

Wilkinson et al: Fc-engineered antibodies with immune effector functions completely abolished

S1 Figure Raw gel images used for preparation of Fig 7.

Samples were analysed on an 8% E-PAGE 48 polyacrylamide gel (Invitrogen Cat. No. EP04808). SeeBlue Plus2 pre-stained molecular weight standards were applied to the reference lanes. The samples were electrophoresed for 25 min on an E-Base electrophoresis device (Invitrogen Cat. No. EB-M03). The gel was stained with 0.03% Coomassie Brilliant Blue R in 10% acetic acid/40% methanol and destained with 8% acetic acid. Images were captured using and E-gel camera (Invitrogen).

Lane images were cut and pasted into the final order to create the images for Fig 7.

Original Gel 1

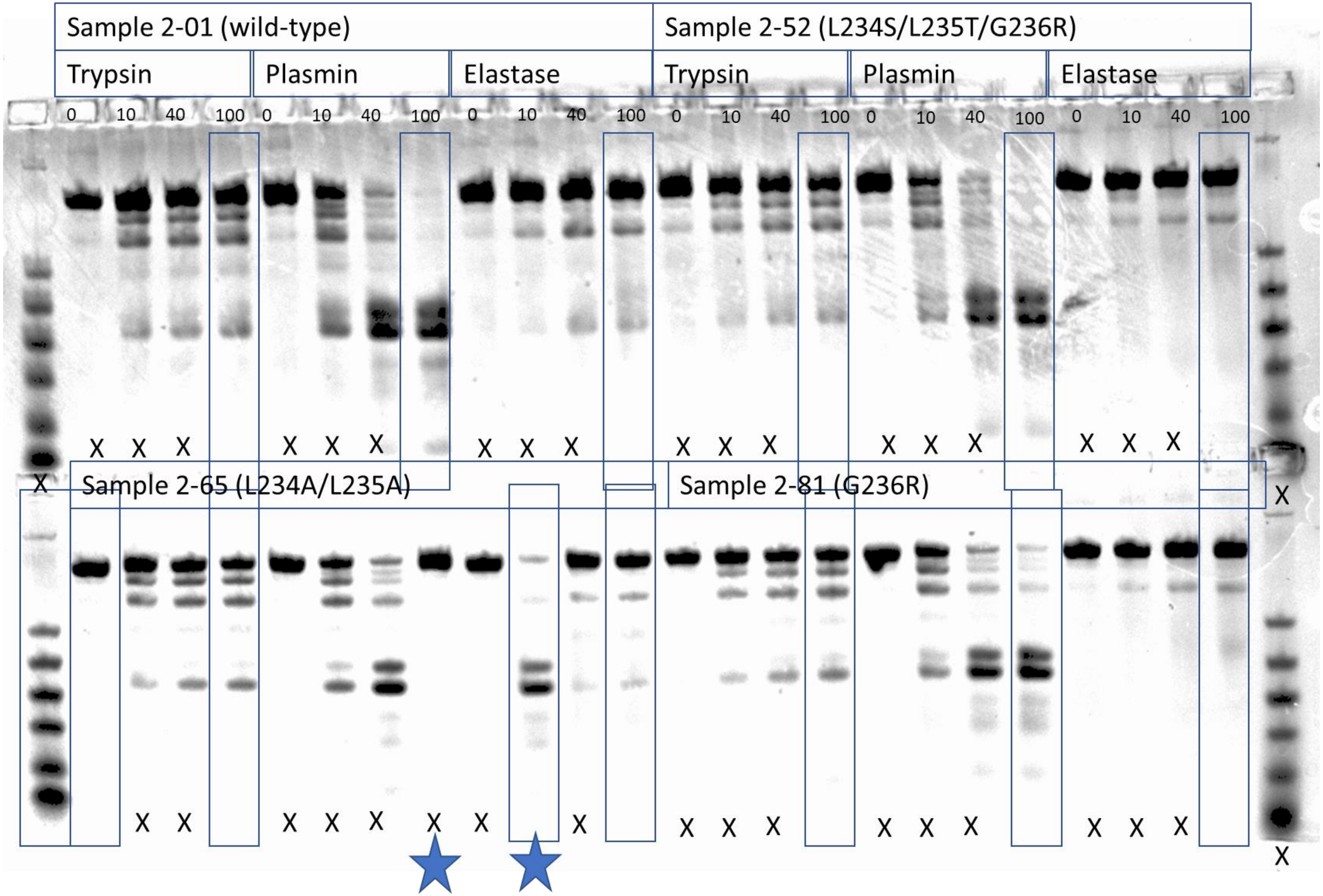

Original Gel 1 Sample list

Lane	Sample	Enzyme	Amount	Lane in Fig 7	Lane	Sample	Enzyme	Amount	Lane in Fig 7
M	markers			X	M	markers			A1
1	2-01	none		X	25	2-65	none		A2
2	2-01	Trypsin	10	X	26	2-65	Trypsin	10	X
3	2-01	Trypsin	40	X	27	2-65	Trypsin	40	X
4	2-01	Trypsin	100	A3	28	2-65	Trypsin	100	A5
5	2-01	none		X	29	2-65	none		X
6	2-01	Elastase	10	X	30	2-65	Elastase	10	X
7	2-01	Elastase	40	X	31	2-65	Elastase	40	X
8	2-01	Elastase	100	A11	32 *	2-65	Plasmin	10	X
9	2-01	none		X	33	2-65	none		X
10	2-01	Plasmin	10	X	34 *	2-65	Elastase	100	A13
11	2-01	Plasmin	40	X	35	2-65	Plasmin	40	X
12	2-01	Plasmin	100	A7	36	2-65	Plasmin	100	A9
13	2-52	none		X	37	2-81	none		X
14	2-52	Trypsin	10	X	38	2-81	Trypsin	10	X
15	2-52	Trypsin	40	X	39	2-81	Trypsin	40	X
16	2-52	Trypsin	100	A4	40	2-81	Trypsin	100	A6
17	2-52	none		X	41	2-81	none		X
18	2-52	Elastase	10	X	42	2-81	Elastase	10	X
19	2-52	Elastase	40	X	43	2-81	Elastase	40	X
20	2-52	Elastase	100	A12	44	2-81	Elastase	100	A14
21	2-52	none		X	45	2-81	none		X
22	2-52	Plasmin	10	X	46	2-81	Plasmin	10	X
23	2-52	Plasmin	40	X	47	2-81	Plasmin	40	X
24	2-52	Plasmin	100	A8	48	2-81	Plasmin	100	A10
M	markers			X	M	markers			X

* the samples originally intended to be loaded in lanes 32 and 34 were switched.

Original Gel 2

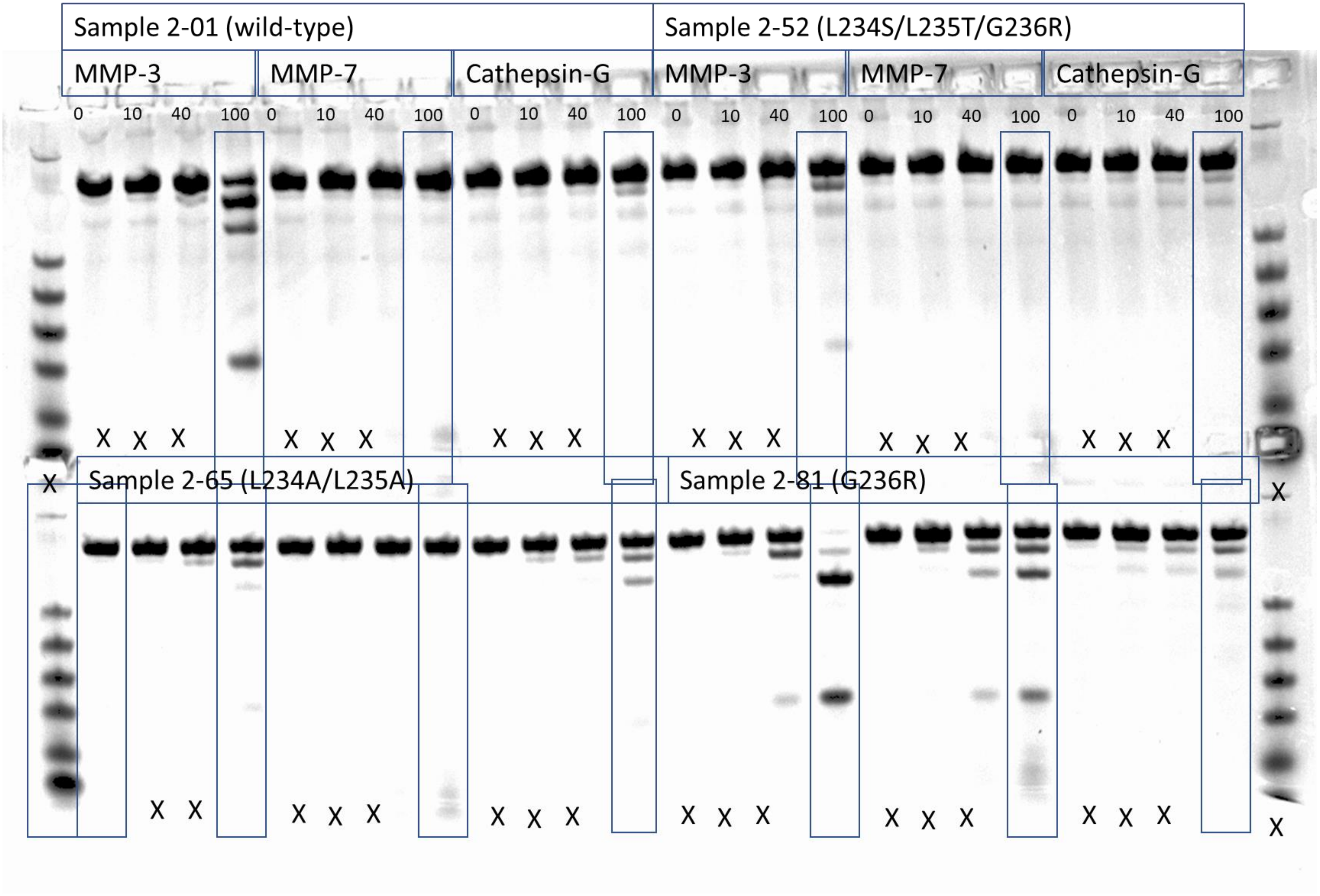

Original Gel 2 Sample list

Lane	Sample	Enzyme	Amount	Lane in Fig 7	Lane	Sample	Enzyme	Amount	Lane in Fig 7
M	markers			X	M	markers			B1
1	2-01	none		X	25	2-65	none		B2
2	2-01	MMP-3	10	X	26	2-65	MMP-3	10	X
3	2-01	MMP-3	40	X	27	2-65	MMP-3	40	X
4	2-01	MMP-3	100	B3	28	2-65	MMP-3	100	B5
5	2-01	none		X	29	2-65	none		X
6	2-01	MMP-7	10	X	30	2-65	MMP-7	10	X
7	2-01	MMP-7	40	X	31	2-65	MMP-7	40	X
8	2-01	MMP-7	100	B15	32 *	2-65	MMP-7	10	B17
9	2-01	none		X	33	2-65	none		X
10	2-01	Cathepsin-G	10	X	34 *	2-65	Cathepsin-G	100	B13
11	2-01	Cathepsin-G	40	X	35	2-65	Cathepsin-G	40	X
12	2-01	Cathepsin-G	100	B11	36	2-65	Cathepsin-G	100	B13
13	2-52	none		X	37	2-81	none		X
14	2-52	MMP-3	10	X	38	2-81	MMP-3	10	X
15	2-52	MMP-3	40	X	39	2-81	MMP-3	40	X
16	2-52	MMP-3	100	B4	40	2-81	MMP-3	100	B6
17	2-52	none		X	41	2-81	none		X
18	2-52	MMP-7	10	X	42	2-81	MMP-7	10	X
19	2-52	MMP-7	40	X	43	2-81	MMP-7	40	X
20	2-52	MMP-7	100	B16	44	2-81	MMP-7	100	B18
21	2-52	none		X	45	2-81	none		X
22	2-52	Cathepsin-G	10	X	46	2-81	Cathepsin-G	10	X
23	2-52	Cathepsin-G	40	X	47	2-81	Cathepsin-G	40	X
24	2-52	Cathepsin-G	100	B12	48	2-81	Cathepsin-G	100	B14
M	markers			X	M	markers			X

Original Gel 3

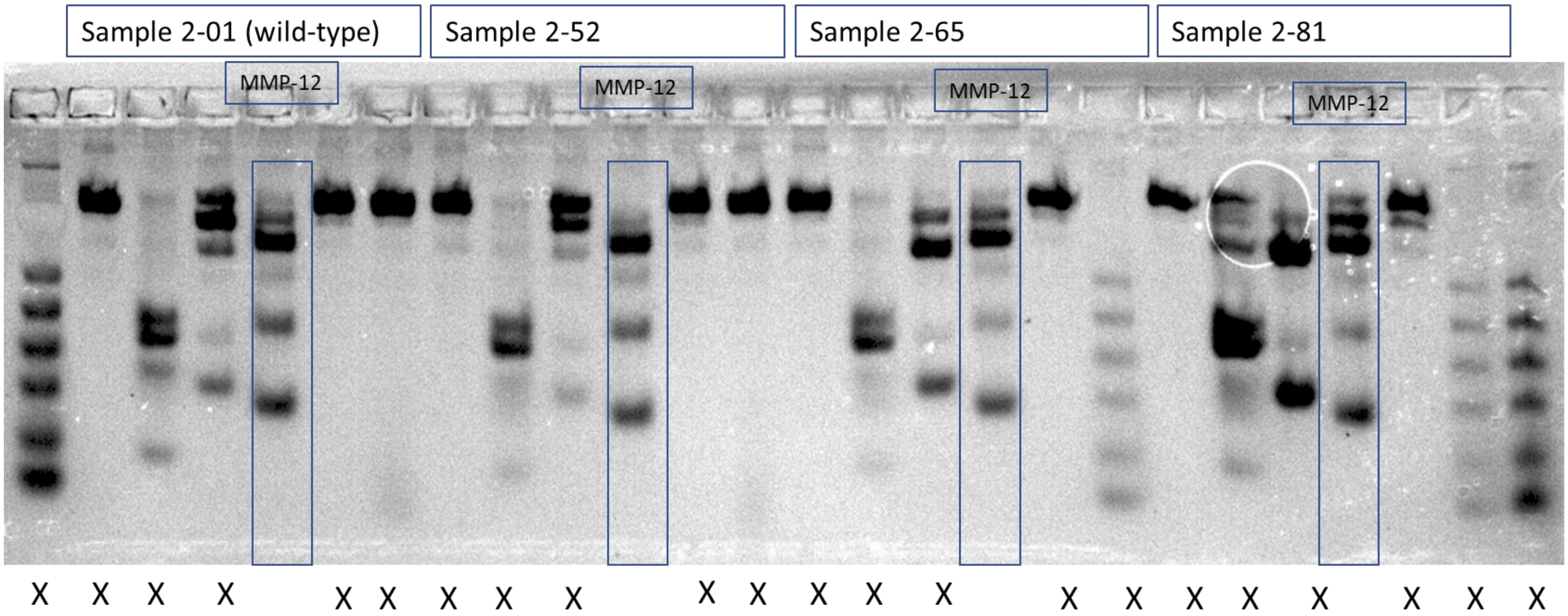

Original Gel 3 Sample list

Lane	Sample	Enzyme	Amount	Lane in Fig 7
M	markers			X
1	2-01	none		X
2	2-01	Elastase	100	X
3	2-01	MMP-3	100	X
4	2-01	MMP-12	100	B7
5	2-01	Cathepsin-G	100	X
6	2-01	MMP-7	100	X
7	2-01	none		X
8	2-01	Elastase	100	X
9	2-01	MMP-3	100	X
10	2-01	MMP-12	100	B8
11	2-01	Cathepsin-G	100	X
12	2-01	MMP-7	100	X
13	2-52	none		X
14	2-52	Elastase	100	X
15	2-52	MMP-3	100	X
16	2-52	MMP-12	100	B9
17	2-52	Cathepsin-G	100	X
18	2-52	MMP-7	100	X
19	2-52	none		X
20	2-52	Elastase	100	X
21	2-52	MMP-3	100	X
22	2-52	MMP-12	100	B10
23	2-52	Cathepsin-G	100	X
24 *	markers			X
M	markers			X

* there was insufficient test sample, so marker was loaded into lane 24
