# Supplementary material for: Fc-engineered antibodies with immune effector functions completely abolished
Source: PLoS One. 2021 Dec 21;16(12):e0260954. doi: 10.1371/journal.pone.0260954 (PMC8691596; doi:10.1371/journal.pone.0260954)
Supplement: S1 Table — (PDF) [file pone.0260954.s002.pdf]

S2 Table: Initial screen of variant antibodies binding to human FcγRI.

Sample	Amino acid alterations	Mean binding response (RU)	Exceed cut-off of 21.9 RU
1-2	L234A/L235A/G236R	30.7	Yes
1-3	L234A/L235D/G236R	28.0	Yes
1-4	L234A/L235E/G236R	31.7	Yes
1-5	L234A/L235G/G236R	27.6	Yes
1-6	L234A/L235H/G236R	23.0	Yes
1-7	L234A/L235I/G236R	28.4	Yes
1-8	L234A/L235K/G236R	34.2	Yes
1-9	L234A/G236R	8.0	
1-10	L234A/L235M/G236R	27.4	Yes
1-11	L234A/L235N/G236R	19.5	
1-12	L234A/L235Q/G236R	34.3	Yes
1-13	L234A/L235R/G236R	26.9	Yes
1-14	L234A/L235S/G236R	32.1	Yes
1-15	L234A/L235T/G236R	8.0	
1-16	L234A/L235V/G236R	25.8	Yes
1-17	L234D/L235A/G236R	29.6	Yes
1-18	L234D/L235D/G236R	22.0	Yes
1-19	L234D/L235E/G236R	22.1	Yes
1-20	L234D/L235G/G236R	27.3	Yes
1-22	L234D/L235I/G236R	25.3	Yes
1-23	L234D/L235K/G236R	20.9	
1-24	L234D/G236R	11.9	
1-25	L234D/L235M/G236R	28.4	Yes
1-26	L234D/L235N/G236R	26.5	Yes
1-27	L234D/L235Q/G236R	8.3	
1-28	L234D/L235R/G236R	47.0	Yes
1-29	L234D/L235S/G236R	26.3	Yes
1-30	L234D/L235T/G236R	4.3	
1-31	L234D/L235V/G236R	27.2	Yes
1-32	L234E/L235A/G236R	44.1	Yes
1-34	L234E/L235E/G236R	30.9	Yes
1-35	L234E/L235G/G236R	71.2	Yes
1-36	L234E/L235H/G236R	21.4	
1-38	L234E/L235K/G236R	34.1	Yes
1-39	L234E/G236R	17.6	
1-40	L234E/L235M/G236R	19.9	
1-41	L234E/L235N/G236R	19.2	
1-42	L234E/L235Q/G236R	33.7	Yes
1-43	L234E/L235R/G236R	26.7	Yes
1-44	L234E/L235S/G236R	39.2	Yes

**Sample**	**Amino acid alterations**	**Mean binding response (RU)**	**Exceed cut-off of 21.9 RU**
1-45	L234E/L235T/G236R	29.6	Yes
1-46	L234E/L235V/G236R	18.4	
1-47	L234G/L235A/G236R	23.0	Yes
1-48	L234G/L235D/G236R	23.6	Yes
1-49	L234G/L235E/G236R	31.8	Yes
1-50	L234G/L235G/G236R	29.5	Yes
1-51	L234G/L235H/G236R	19.0	
1-52	L234G/L235I/G236R	29.2	Yes
1-53	L234G/L235K/G236R	27.4	Yes
1-54	L234G/G236R	28.5	Yes
1-55	L234G/L235M/G236R	23.2	Yes
1-56	L234G/L235N/G236R	26.8	Yes
1-57	L234G/L235Q/G236R	13.6	
1-58	L234G/L235R/G236R	29.3	Yes
1-59	L234G/L235S/G236R	21.8	
1-60	L234G/L235T/G236R	26.5	Yes
1-61	L234G/L235V/G236R	32.9	Yes
1-62	L234H/L235A/G236R	29.4	Yes
1-63	L234H/L235D/G236R	38.5	Yes
1-64	L234H/L235E/G236R	22.8	Yes
1-65	L234H/L235G/G236R	31.6	Yes
1-66	L234H/L235H/G236R	36.3	Yes
1-67	L234H/L235I/G236R	2.8	
1-68	L234H/L235K/G236R	29.2	Yes
1-69	L234H/G236R	22.9	Yes
1-70	L234H/L235M/G236R	31.3	Yes
1-71	L234H/L235N/G236R	24.6	Yes
1-72	L234H/L235Q/G236R	33.3	Yes
1-73	L234H/L235R/G236R	35.3	Yes
1-74	L234H/L235S/G236R	10.9	
1-75	L234H/L235T/G236R	44.9	Yes
1-76	L234H/L235V/G236R	25.6	Yes
1-77	L234K/L235A/G236R	23.0	Yes
1-78	L234K/L235D/G236R	26.6	Yes
1-79	L234K/L235E/G236R	29.9	Yes
1-80	L234K/L235G/G236R	27.4	Yes
1-81	L234K/L235H/G236R	25.3	Yes
1-82	L234K/L235I/G236R	30.9	Yes
1-83	L234K/L235K/G236R	37.6	Yes
1-84	L234K/G236R	31.2	Yes
1-85	L234K/L235M/G236R	23.9	Yes

Sample	Amino acid alterations	Mean binding response (RU)	Exceed cut-off of 21.9 RU
1-86	L234K/L235N/G236R	24.8	Yes
1-87	L234K/L235Q/G236R	8.4	
1-88	L234K/L235R/G236R	13.9	
1-89	L234K/L235S/G236R	16.1	
1-90	L234K/L235T/G236R	15.8	
1-91	L234K/L235V/G236R	12.8	
1-92	L234N/L235A/G236R	22.3	Yes
1-93	L234N/L235D/G236R	13.4	
1-94	L234N/L235E/G236R	16.4	
1-95	L234N/L235G/G236R	22.1	Yes
1-96	L234N/L235H/G236R	17.7	
1-97	L234N/L235I/G236R	14.1	
1-98	L234N/L235K/G236R	25.0	Yes
1-99	L234N/G236R	22.3	Yes
1-100	L234N/L235M/G236R	19.9	
1-101	L234N/L235N/G236R	16.9	
1-102	L234N/L235Q/G236R	4.5	
1-103	L234N/L235R/G236R	30.4	Yes
1-104	L234N/L235S/G236R	18.4	
1-105	L234N/L235T/G236R	8.6	
1-106	L234N/L235V/G236R	18.9	
1-107	L234Q/L235A/G236R	13.4	
1-108	L234Q/L235D/G236R	18.9	
1-109	L234Q/L235E/G236R	28.8	Yes
1-110	L234Q/L235G/G236R	28.1	Yes
1-111	L234Q/L235H/G236R	10.0	
1-112	L234Q/L235I/G236R	34.7	Yes
1-113	L234Q/L235K/G236R	26.9	Yes
1-115	L234Q/L235M/G236R	38.4	Yes
1-116	L234Q/L235N/G236R	26.8	Yes
1-117	L234Q/L235Q/G236R	16.8	
1-118	L234Q/L235R/G236R	20.1	
1-119	L234Q/L235S/G236R	11.9	
1-120	L234Q/L235T/G236R	20.0	
1-121	L234Q/L235V/G236R	20.9	
1-122	L234R/L235A/G236R	28.1	Yes
1-123	L234R/L235D/G236R	11.1	
1-124	L234R/L235E/G236R	16.7	
1-125	L234R/L235G/G236R	31.9	Yes
1-126	L234R/L235H/G236R	14.3	
1-127	L234R/L235I/G236R	10.7	

Sample	Amino acid alterations	Mean binding response (RU)	Exceed cut-off of 21.9 RU
1-128	L234R/L235K/G236R	19.2	
1-129	L234R/G236R	21.1	
1-131	L234R/L235N/G236R	10.8	
1-132	L234R/L235Q/G236R	10.1	
1-133	L234R/L235R/G236R	17.2	
1-134	L234R/L235S/G236R	34.0	Yes
1-136	L234R/L235V/G236R	32.5	Yes
1-137	L234S/L235A/G236R	29.6	Yes
1-139	L234S/L235E/G236R	11.6	
1-140	L234S/L235G/G236R	11.2	
1-142	L234S/L235I/G236R	12.0	
1-143	L234S/L235K/G236R	23.8	Yes
1-145	L234S/L235M/G236R	32.0	Yes
1-146	L234S/L235N/G236R	22.7	Yes
1-147	L234S/L235Q/G236R	29.6	Yes
1-148	L234S/L235R/G236R	11.1	
1-149	L234S/L235S/G236R	25.6	Yes
1-150	L234S/L235T/G236R	16.2	
1-152	L234T/L235A/G236R	11.8	
1-153	L234T/L235D/G236R	12.2	
1-154	L234T/L235E/G236R	24.0	Yes
1-155	L234T/L235G/G236R	26.9	Yes
1-156	L234T/L235H/G236R	18.9	
1-157	L234T/L235I/G236R	14.0	
1-158	L234T/L235K/G236R	6.7	
1-160	L234T/L235M/G236R	31.6	Yes
1-161	L234T/L235N/G236R	22.4	Yes
1-162	L234T/L235Q/G236R	17.0	
1-163	L234T/L235R/G236R	13.6	
1-164	L234T/L235S/G236R	25.4	Yes
1-167	L234A/L235A (LALA)	65.0	Yes

Samples of undiluted culture supernatants were injected over immobilised FcγRI for 1 min at 30 µL/min and allowed to disassociate for 2 min. The whole experiment was repeated three times. Due to the relatively crude nature of the samples, some sensorgrams were unsatisfactory and were eliminated from analysis. The mean and standard deviation of the specific binding response was calculated for each sample. The mean of all standard deviations was 6.7 RU. Sample 1-119 (L234Q/L235S/G236R) was taken as a representative variant which was effectively silenced. It gave a mean response of 11.9 RU. A cut-off of 21.9 RU was calculated as equal to the mean response of this representative variant plus 1.645 times the mean standard deviation of the samples. Samples giving a response above this cut-off were deemed to give significantly greater binding to FcγRI compared with the representative sample 1-119.
