# Supplementary material for: Fc-engineered antibodies with immune effector functions completely abolished
Source: PLoS One. 2021 Dec 21;16(12):e0260954. doi: 10.1371/journal.pone.0260954 (PMC8691596; doi:10.1371/journal.pone.0260954)
Supplement: S2 Table — (PDF) [file pone.0260954.s003.pdf]

S3 Table: Individual results for Table 2: Binding responses of CD20, CD3 and CD52 antibodies binding to Fc gamma receptors.

Sample	Specificity	Amino acid alterations and sample description	human FcγRI Rep 1	human FcγRI Rep 2	human FcγRI Mean	human FcγRI SD	human FcγRIIa R131 Rep 1	human FcγRIIa R131 Rep 2	human FcγRIIa R131 Mean	human FcγRIIa R131 SD	human FcγRIIb Rep 1	human FcγRIIb Rep 2	human FcγRIIb Mean	human FcγRIIb SD	human FcγRIIIa V158 Rep 1	human FcγRIIIa V158 Rep 2	human FcγRIIIa V158 Mean	human FcγRIIIa V158 SD	human FcγRIIIa F158 Rep 1	human FcγRIIIa F158 Rep 2	human FcγRIIIa F158 Mean	human FcγRIIIa F158 SD	human FcγRIIIb NA2 Rep 1	human FcγRIIIb NA2 Rep 2	human FcγRIIIb NA2 Mean	human FcγRIIIb NA2 SD	mouse FcγRI Rep 1	mouse FcγRI Rep 2	mouse FcγRI Mean	mouse FcγRI SD	rat FcγRI Rep 1	rat FcγRI Rep 2	rat FcγRI Mean	rat FcγRI SD
PBS			-0.4	0.8	0.2	0.9	-8.4	-2.1	-5.2	4.4	-7.4	-1.2	-4.3	4.4	0.3	-6.7	-3.2	4.9	-4.6	-1.6	-3.1	2.1	-4.7	5*	-4.7	na	0.1	265.7*	0.1	na	0.3	44.5*	0.3	na
PBS			1.1	1.3	1.2	0.2	7.0	0.3	3.7	4.7	6.0	1.7	3.9	3.1	0.4	0.7	0.5	0.2	4.1	0.7	2.4	2.4	3.9	0.0	1.9	2.7	0.1	2.8	1.5	1.9	1.6	-3.2	-0.8	3.3
PBS			-0.9	0.9	0.0	1.2	1.4	-0.7	0.3	1.5	1.4	0.2	0.8	0.8	-0.8	-0.4	-0.6	0.3	0.4	-0.2	0.1	0.4	0.9	-0.1	0.4	0.7	-0.3	-2.6	-1.4	1.6	-1.9	-0.1	-1.0	1.3
PBS			5.3	-117.1*	5.3	na	1.1	-2.0	-0.4	2.2	1.3	-4.4	-1.5	4.0	-2.4	1.1	-0.6	2.4	0.1	-3.0	-1.5	2.2	0.4	-4.0	-1.8	3.1	0.0	3.4	1.7	2.4	-0.3	1.2	0.4	1.1
PBS			3.5	-1.8	0.9	3.8	0.9	-0.4	0.3	0.9	1.5	-1.3	0.1	2.0	-2.1	-0.7	-1.4	1.0	1.6	-0.5	0.6	1.5	0.6	-0.8	-0.1	1.0	8.3	-0.8	3.8	6.5	0.8	-1.1	-0.1	1.4
PBS			0.9	3.2	2.0	1.6	7.8	1.2	4.5	4.6	8.9	1.3	5.1	5.4	-4.8	nd	-4.8	na	7.6	1.1	4.4	4.6	8.4	1.1	4.8	5.1	0.1	nd	0.1	na	-2.8	nd	-2.8	na
PBS			4.8	-1.4	1.7	4.4	1.5	-0.8	0.4	1.6	2.1	nd	2.1	na	-5.3	nd	-5.3	na	2.4	-0.6	0.9	2.1	1.2	-0.4	0.4	1.1	-1.0	nd	-1.0	na	-1.5	nd	-1.5	na
Mean of PBS negative controls					1.3				0.5				0.8				-1.7				0.5				0.5				0.9				-0.6	
SD					2.3				3.9				4.1				2.6				3.0				3.2				3.0				1.6	
Cut point = Mean+1.645*SD					5.1				6.8				7.5				2.5				5.4				5.8				5.8				2.0	
2-1	CD20	wild type reference	2487.8	2237.8	2362.8	176.8	144.4	131.4	137.9	9.2	53.4	50.4	51.9	2.2	652.8	637.8	645.3	10.7	390.4	357.5	373.9	23.3	205.5	208.2	206.9	1.9	1981.1	1889.4	1935.3	64.8	815.7	761.8	788.7	38.2
2-19	CD20	L234G/L235S/G236R	-1.2	-1.8	-1.5	0.4	5.0	-0.8	2.1	4.1	2.7	-2.8	-0.1	3.9	-0.4	0.8	0.2	0.8	3.3	-1.4	1.0	3.3	0.0	-3.1	-1.6	2.2	-3.2	-1.7	-2.5	1.1	-7.2	-1.0	-4.1	4.4
2-52	CD20	L234S/L235T/G236R	-2.2	-0.5	-1.3	1.2	6.1	-0.9	2.6	4.9	3.7	-2.7	0.5	4.5	-4.6	-3.4	-4.0	0.9	5.0	-1.1	1.9	4.3	11.0*	-3.0	-3.0	na	-1.8	-6.6	-4.2	3.3	0.0	-3.8	-1.9	2.7
2-53	CD20	L234S/L235V/G236R	-2.7	-0.5	-1.6	1.5	2.5	-1.9	0.3	3.1	1.8	-3.5	-0.8	3.7	-0.3	0.7	0.2	0.7	2.7	-2.8	0.0	3.9	4.7	-3.6	0.6	5.9	1.3	-1.7	-0.2	2.2	0.1	-1.2	-0.5	0.9
2-60	CD20	L234T/L235Q/G236R	-2.4	-1.4	-1.9	0.7	2.4	-2.1	0.2	3.2	1.6	-3.8	-1.1	3.8	-2.0	-1.7	-1.8	0.2	1.4	-2.5	-0.5	2.8	1.3	-4.2	-1.5	3.9	-2.2	-3.7	-3.0	1.1	-1.2	-3.0	-2.1	1.3
2-63	CD20	L234T/L235T/G236R	-3.4	-1.9	-2.7	1.1	4.4	-0.7	1.9	3.6	3.5	-2.3	0.6	4.1	-0.3	3.2	1.4	2.5	4.5	-1.3	1.6	4.1	2.2	-2.4	-0.1	3.3	-1.1	0.0	-0.6	0.8	-0.4	-0.5	-0.4	0.0
2-65	CD20	L234A/L235A (LALA)	606.3	501.4	553.9	74.2	14.5	11.6	13.1	2.0	5.8	9.1	7.5	2.4	73.5	53.1	63.3	14.4	29.4	28.6	29.0	0.5	9.2	13.1	11.2	2.8	-10.9	-13.6	-12.3	1.9	-7.8	-2.7	-5.3	3.6
2-66	CD20	L234A/L235A/P329G (LALAPG)	48.1	14.7*	48.1	na	2.6	3.4	3.0	0.6	6.4	1.8	4.1	3.3	0.7	1.5	1.1	0.6	6.3	3.1	4.7	2.3	9.3	0.8	5.1	6.0	2.2	2.2	2.2	0.0	2.6	-1.4	0.6	2.8
2-67	CD20	N297Q (aglycosyl)	499.8	358.2*	499.8	na	6.3	3.9	5.1	1.6	5.4	-0.6	2.4	4.3	1.1	0.9	1.0	0.1	3.4	1.5	2.4	1.3	6.8	-1.4	2.7	5.7	67.3	53.8	60.5	9.5	579.8	540.9	560.3	27.5
3-1	CD3	wild type reference	2500.2	2310.3	2405.3	134.3	125.4	127.4	126.4	1.4	42.5	44.9	43.7	1.6	699.9	679.0	689.5	14.8	266.9	252.8	259.9	9.9	142.1	147.2	144.6	3.6	2049.0	1939.0	1994.0	77.8	821.5	756.7	789.1	45.8
3-19	CD3	L234G/L235S/G236R	-2.9	-7.6	-5.2	3.3	-5.1	-4.6	-4.8	0.3	-5.4	-4.7	-5.1	0.5	-8.9	-12.7	-10.8	2.7	-5.1	-4.3	-4.7	0.6	-6.5	-6.3	-6.4	0.1	-3.1	-4.9	-4.0	1.3	-2.4	-4.4	-3.4	1.4
3-52	CD3	L234S/L235T/G236R	-7.4	-3.1	-5.2	3.0	2.8	0.0	1.4	2.0	7.2	0.6	3.9	4.7	-5.6	-9.2	-7.4	2.5	6.1	0.5	3.3	3.9	8.2	1.2	4.7	4.9	-5.0	-5.9	-5.4	0.7	-3.6	-5.0	-4.3	1.0
3-53	CD3	L234S/L235V/G236R	-5.7	-5.0	-5.4	0.4	-4.4	-2.4	-3.4	1.4	-5.0	-3.3	-4.2	1.2	-7.6	-10.6	-9.1	2.2	-4.0	-2.8	-3.4	0.9	-5.5	-4.1	-4.8	1.0	-5.1	-7.3	-6.2	1.6	-1.7	-6.3	-4.0	3.2
3-60	CD3	L234T/L235Q/G236R	-3.5	-2.9	-3.2	0.4	1.9	-2.3	-0.2	3.0	-0.7	-4.9	-2.8	3.0	-5.8	-9.5	-7.6	2.7	-0.1	-1.8	-0.9	1.2	-2.3	-3.7	-3.0	0.9	-3.0	-5.3	-4.1	1.6	0.3	-3.9	-1.8	3.0
3-63	CD3	L234T/L235T/G236R	-2.8	-3.5	-3.1	0.4	-2.2	-3.3	-2.8	0.8	-2.2	-4.3	-3.3	1.5	-6.4	-9.2	-7.8	2.0	-0.5	-3.4	-2.0	2.1	-3.6	-5.1	-4.4	1.0	-5.5	-4.6	-5.1	0.6	-2.7	-3.9	-3.3	0.9
3-65	CD3	L234A/L235A (LALA)	540.8	528.3	534.5	8.9	9.4	2.1	5.8	5.1	2.5	-3.0	-0.3	3.9	70.6	63.8	67.2	4.8	17.4	12.3*	17.4	na	2.6	4.7	3.7	1.5	-0.7	-2.4	-1.6	1.2	-3.0	-5.3	-4.1	1.6
3-66	CD3	L234A/L235A/P329G (LALAPG)	44.2	34.7	39.4	6.8	-4.4	-5.6	-5.0	0.8	-4.3	-6.9	-5.6	1.8	-7.8	-10.3	-9.1	1.7	-1.9	-5.7	-3.8	2.7	6.1	-4.0	1.1	7.2	-5.2	-7.6	-6.4	1.7	-3.8	-6.8	-5.3	2.1
3-67	CD3	N297Q (aglycosyl)	501.9	502.1	502.0	0.1	5.9	-2.6	1.7	6.0	-1.8	-7.5	-4.7	4.0	-10.6	-11.5	-11.1	0.7	-0.7	-5.9	-3.3	3.7	0.9	-6.5	-2.8	5.2	47.4	42.7	45.0	3.3	487.0	471.0	479.0	11.3
4-1	CD52	wild type reference	2267.5	1951.9	2109.7	223.2	129.0	125.6	127.3	2.4	46.3	40.5	43.4	4.1	526.4	406.2	466.3	85.0	315.9	276.4	296.1	27.9	176.6	174.5	175.5	1.5	1675.7	1565.1	1620.4	78.1	608.5	577.9	593.2	21.7
4-19	CD52	L234G/L235S/G236R	-2.3	-4.7	-3.5	1.7	-3.4	-3.6	-3.5	0.2	-3.0	-8.0	-5.5	3.5	-4.3	-1.0	-2.6	2.3	-3.5	-5.0	-4.2	1.0	-3.5	-6.1	-4.8	1.9	-1.6	-3.3	-2.4	1.2	-0.6	-2.9	-1.7	1.6
4-52	CD52	L234S/L235T/G236R	-4.0	-6.1	-5.1	1.5	-1.0	-5.1	-3.1	2.9	-1.7	-7.1	-4.4	3.9	-5.0	-2.7	-3.8	1.7	-1.1	-5.5	-3.3	3.2	-1.8	-6.7	-4.3	3.4	-2.4	-5.1	-3.7	1.9	-1.4	-3.6	-2.5	1.5
4-53	CD52	L234S/L235V/G236R	-21.6	-18.9	-20.3	2.0	-2.0	-6.5	-4.2	3.1	6.1	-1.2	2.4	5.2	-3.9	0.4	-1.8	3.0	5.7	-1.3	2.2	4.9	5.2	0.0	2.6	3.7	-2.6	-2.9	-2.8	0.2	-2.6	-1.9	-2.2	0.4
4-60	CD52	L234T/L235Q/G236R	-11.9	-9.4	-10.6	1.8	-1.9	-2.5	-2.2	0.4	3.3	-0.7	1.3	2.9	-1.8	-0.4	-1.1	0.9	2.4	-1.1	0.7	2.5	2.6	-1.9	0.4	3.2	2.7	-3.3	-0.3	4.2	3.8	-2.0	0.9	4.1
4-63	CD52	L234T/L235T/G236R	-22.7	-18.6	-20.7	2.9	-10.5	-6.9	-8.7	2.6	0.2	-0.3	-0.1	0.3	-3.3	1.1	-1.1	3.1	-2.5*	-1.3	-1.3	na	13.7*	0.8	0.8	na	-3.9	-3.0	-3.4	0.7	-3.3	-3.1	-3.2	0.2
4-65	CD52	L234A/L235A (LALA)	483.5	419.4	451.5	45.3	7.2	4.4	5.8	1.9	2.3	2.3	2.3	0.0	69.3	58.3	63.8	7.8	17.1	16.9	17.0	0.2	7.1	4.6	5.9	1.8	-0.2	4.4	2.1	3.2	-3.0	1.9	-0.6	3.4
4-66	CD52	L234A/L235A/P329G (LALAPG)	35.7	31.8	33.7	2.7	1.0	1.4	1.2	0.3	2.5	3.3	2.9	0.6	-3.0	nd	-3.0	na	2.7	3.2	3.0	0.3	4.5	2.3	3.4	1.5	-2.5	1.7	-0.4	3.0	-1.7	1.6	-0.1	2.4
4-67	CD52	N297Q (aglycosyl)	458.3	402.9	430.6	39.2	0.2	5.9	3.1	4.0	0.7	5.0	2.9	3.0	-3.4	-7.5	-5.5	2.9	0.0	5.6	2.8	4.0	0.9	4.5	2.7	2.5	54.1	48.8	51.5	3.8	506.7	470.4	488.5	25.7

nd = sample not analysed
na = not applicable
* = point dropped due to unsatisfactory sensorgram (e.g. air bubble)
highlight = result above cut point
