# Supplementary material for: Fc-engineered antibodies with immune effector functions completely abolished
Source: PLoS One. 2021 Dec 21;16(12):e0260954. doi: 10.1371/journal.pone.0260954 (PMC8691596; doi:10.1371/journal.pone.0260954)
Supplement: S3 Table — (PDF) [file pone.0260954.s004.pdf]

S4 Table: Mean and SD of absolute absorbance responses for Table 4: Mean normalised absorbance of antibodies binding human C1q

Target antigen	Amino acid alterations and sample description	Expt 1 Plate 1 Mean	Expt 1 Plate 1 SD	Expt 2 Plate 2 Mean	Expt 2 Plate 2 SD	Expt 3 Plate 3 Mean	Expt 3 Plate 3 SD	Expt 3 Plate 4 Mean	Expt 3 Plate 4 SD
	PBS	0.050	0.020	0.073	0.037	0.111	0.017	0.070	0.023
	PBS	nd	nd	nd	nd	0.081	0.007	0.061	0.022
	PBS	nd	nd	nd	nd	0.092	0.009	0.041	0.003
CD20	wild type reference	2.484	0.208	2.773	0.131	2.187	0.157	2.237	0.152
CD20	L234G/L235S/G236R	0.085	0.009	0.020	0.011	0.106	0.016	0.077	0.004
CD20	L234S/L235T/G236R	0.065	0.003	0.029	0.014	0.080	0.019	0.078	0.005
CD20	L234S/L235V/G236R	0.080	0.011	0.064	0.020	0.089	0.026	0.090	0.022
CD20	L234T/L235Q/G236R	0.066	0.010	0.031	0.027	0.086	0.013	0.068	0.020
CD20	L234T/L235T/G236R	0.064	0.003	0.073	0.049	0.088	0.015	0.065	0.012
CD20	L234A/L235A (LALA)	nd	nd	nd	nd	0.080	0.026	0.075	0.002
CD20	L234A/L235A/P329G (LALAPG reference)	0.086	0.017	0.090	0.059	0.111	0.006	0.092	0.009
CD20	N297Q (aglycosyl)	nd	nd	nd	nd	0.078	0.007	0.074	0.004
		**Expt 4 Plate 5 Mean**	**Expt 4 Plate 5 SD**	**Expt 4 Plate 6 Mean**	**Expt 4 Plate 6 SD**	**Expt 5 Plate 7 Mean**	**Expt 5 Plate 7 SD**	**Expt 5 Plate 8 Mean**	**Expt 5 Plate 8 SD**
	PBS	0.050	0.002	0.050	0.002	0.038	0.002	0.039	0.002
	PBS	0.053	0.005	0.048	0.003	0.043	0.003	0.047	0.002
	PBS	0.059	0.009	0.049	0.000	0.055	0.012	0.050	0.006
	PBS	0.051	0.002	0.052	0.003	0.049	0.004	0.049	0.008
	PBS	0.053	0.003	0.054	0.007	0.049	0.009	0.054	0.003
CD3	wild type reference	0.547	0.027	0.991	0.080	0.409	0.026	0.456	0.049
CD3	L234G/L235S/G236R	0.050	0.002	0.055	0.003	0.046	0.001	0.052	0.002
CD3	L234S/L235T/G236R	0.046	0.001	0.049	0.002	0.041	0.005	0.051	0.003
CD3	L234S/L235V/G236R	0.047	0.002	0.047	0.000	0.039	0.001	0.044	0.002
CD3	L234T/L235Q/G236R	0.046	0.001	0.048	0.003	0.043	0.003	0.047	0.003
CD3	L234T/L235T/G236R	0.047	0.001	0.048	0.001	0.047	0.008	0.049	0.002
CD3	L234A/L235A (LALA)	0.052	0.002	0.054	0.001	0.051	0.013	0.050	0.002
CD3	L234A/L235A/P329G (LALAPG reference)	0.054	0.006	0.049	0.002	0.041	0.003	0.048	0.003
CD3	N297Q (aglycosyl)	0.053	0.003	0.051	0.001	0.050	0.011	0.051	0.003
CD52	wild type reference	1.166	0.075	1.680	0.086	0.624	0.098	0.674	0.092
CD52	L234G/L235S/G236R	0.050	0.003	0.057	0.002	0.056	0.001	0.056	0.001
CD52	L234S/L235T/G236R	0.049	0.001	0.055	0.002	0.051	0.001	0.056	0.004
CD52	L234S/L235V/G236R	0.053	0.005	0.061	0.003	0.058	0.007	0.060	0.009
CD52	L234T/L235Q/G236R	0.052	0.002	0.059	0.002	0.049	0.002	0.055	0.005
CD52	L234T/L235T/G236R	0.055	0.001	0.060	0.003	0.059	0.007	0.057	0.003
CD52	L234A/L235A (LALA)	0.058	0.001	0.066	0.002	0.054	0.003	0.061	0.002
CD52	L234A/L235A/P329G (LALAPG reference)	0.053	0.002	0.064	0.005	0.059	0.001	0.056	0.003
CD52	N297Q (aglycosyl)	0.049	0.002	0.053	0.002	0.037	0.001	0.050	0.002

means and standard deviations are each of three replicates
nd = sample not done
