# Supplementary material for: Fc-engineered antibodies with immune effector functions completely abolished
Source: PLoS One. 2021 Dec 21;16(12):e0260954. doi: 10.1371/journal.pone.0260954 (PMC8691596; doi:10.1371/journal.pone.0260954)
Supplement: S4 Table — (PDF) [file pone.0260954.s005.pdf]

S5 Table: Individual results for Table 5: Mean normalised binding of antibodies to FcRn

Sample	Specificity	Amino acid alterations and sample description	human FcRn Rep 1	human FcRn Rep 2	human FcRn Mean	human FcRn SD
PBS			0.0	0.2	0.1	0.1
2-1	CD20	wild type reference	100.0	100.0	100.0	na
2-19	CD20	L234G/L235S/G236R	106.1	97.1	101.6	6.3
2-52	CD20	L234S/L235T/G236R	101.8	100.4	101.1	1.0
2-53	CD20	L234S/L235V/G236R	99.9	109.8	104.9	7.0
2-60	CD20	L234T/L235Q/G236R	99.7	98.5	99.1	0.9
2-63	CD20	L234T/L235T/G236R	91.0	101.6	96.3	7.5
2-65	CD20	L234A/L235A (LALA)	97.4	94.9	96.1	1.8
2-66	CD20	L234A/L235A/P329G (LALAPG)	99.8	97.0	98.4	1.9
2-67	CD20	N297Q (aglycosyl)	87.2	86.2	86.7	0.7
3-1	CD3	wild type reference	100.0	100.0	100.0	na
3-19	CD3	L234G/L235S/G236R	105.6	99.2	102.4	4.5
3-52	CD3	L234S/L235T/G236R	107.6	100.3	103.9	5.2
3-53	CD3	L234S/L235V/G236R	104.2	97.4	100.8	4.8
3-60	CD3	L234T/L235Q/G236R	104.0	97.7	100.8	4.4
3-63	CD3	L234T/L235T/G236R	102.5	97.2	99.9	3.7
3-65	CD3	L234A/L235A (LALA)	98.8	94.7	96.7	3.0
3-66	CD3	L234A/L235A/P329G (LALAPG)	96.2	91.2	93.7	3.5
3-67	CD3	N297Q (aglycosyl)	87.5	82.6	85.1	3.5
4-1	CD52	wild type reference	100.0	100.0	100.0	na
4-19	CD52	L234G/L235S/G236R	123.7	111.7	117.7	8.5
4-52	CD52	L234S/L235T/G236R	121.5	110.0	115.7	8.1
4-53	CD52	L234S/L235V/G236R	125.3	112.9	119.1	8.8
4-60	CD52	L234T/L235Q/G236R	120.7	108.8	114.8	8.4
4-63	CD52	L234T/L235T/G236R	123.0	113.7	118.3	6.6
4-65	CD52	L234A/L235A (LALA)	109.2	100.4	104.8	6.2
4-66	CD52	L234A/L235A/P329G (LALAPG)	106.1	103.3	104.7	2.0
4-67	CD52	N297Q (aglycosyl)	109.9	96.7	103.3	9.3

na = not applicable (data are normalised to 100% for wild-type reference)
