# Supplementary material for: Fc-engineered antibodies with immune effector functions completely abolished
Source: PLoS One. 2021 Dec 21;16(12):e0260954. doi: 10.1371/journal.pone.0260954 (PMC8691596; doi:10.1371/journal.pone.0260954)
Supplement: S5 Table — (PDF) [file pone.0260954.s006.pdf]

S6 Table: Yield and purity of variant antibodies produced from HEK cells

Sample	Amino acid alterations and sample description	Yield (mg)	Yield (relative to wt)	Purity by SEC-HPLC (%)
2-1	wild type	2.6	1.00	97.7
2-2	L234A/L235A/G236R	2.6	1.14	97.6
2-3	L234A/G236R	2.8	1.00	97.7
2-4	L234A/L235S/G236R	2.5	0.94	98.0
2-5	L234A/L235T/G236R	2.6	0.98	97.7
2-6	L234D/L235H/G236R	2.9	1.21	97.6
2-7	L234D/L235K/G236R	2.6	1.28	97.4
2-8	L234D/G236R	3.0	1.75	97.8
2-9	L234D/L235Q/G236R	2.8	1.45	97.8
2-10	L234D/L235S/G236R	2.5	0.97	97.1
2-11	L234D/L235T/G236R	3.1	1.45	97.6
2-12	L234E/L235D/G236R	2.7	1.13	97.8
2-13	L234E/L235H/G236R	2.9	1.07	97.7
2-14	L234E/L235I/G236R	2.4	1.15	97.8
2-15	L234E/G236R	2.6	1.06	97.9
2-16	L234E/L235V/G236R	2.4	1.00	97.9
2-17	L234G/L235H/G236R	2.5	1.08	98.1
2-18	L234G/L235Q/G236R	2.6	1.16	97.8
2-19	L234G/L235S/G236R	3.0	1.19	98.1
2-20	L234H/L235I/G236R	2.9	1.33	97.5
2-21	L234H/L235S/G236R	2.6	1.07	98.2
2-22	L234K/L235Q/G236R	1.6	0.67	98.1
2-23	L234K/L235R/G236R	2.6	0.65	98.2
2-24	L234K/L235S/G236R	1.9	0.69	98.4
2-25	L234K/L235T/G236R	2.2	0.81	97.6
2-26	L234K/L235V/G236R	1.8	0.71	98.1
2-27	L234Q/L235A/G236R	2.0	0.57	98.1
2-28	L234Q/L235D/G236R	1.4	0.49	95.7
2-29	L234Q/L235H/G236R	1.8	0.54	98.0
2-30	L234Q/G236R	1.8	0.43	98.0
2-31	L234Q/L235Q/G236R	1.5	0.53	95.8
2-32	L234Q/L235R/G236R	1.5	0.60	98.3
2-33	L234Q/L235S/G236R	1.4	0.55	97.8
2-34	L234Q/L235T/G236R	1.9	0.72	97.9
2-35	L234Q/L235V/G236R	1.6	0.68	97.3
2-36	L234R/L235D/G236R	2.0	0.71	97.4
2-37	L234R/L235E/G236R	2.0	0.73	98.2
2-38	L234R/L235H/G236R	1.8	0.57	98.1
2-39	L234R/L235I/G236R	1.8	0.55	98.4
2-40	L234R/L235K/G236R	1.5	0.46	98.6
2-41	L234R/G236R	1.8	0.57	98.2
2-42	L234R/L235Q/G236R	1.9	0.65	98.4
2-43	L234R/L235R/G236R	2.2	0.85	98.4
2-44	L234R/L235T/G236R	1.9	0.69	98.4
2-45	L234S/L235D/G236R	2.5	0.96	98.0
2-46	L234S/L235E/G236R	2.4	0.86	98.0
2-47	L234S/L235G/G236R	2.3	0.82	97.7
2-48	L234S/L235H/G236R	2.6	1.16	98.2
2-49	L234S/L235I/G236R	2.7	1.14	98.0
2-50	L234S/G236R	3.0	1.39	98.0
2-51	L234S/L235R/G236R	2.7	1.31	97.8
2-52	L234S/L235T/G236R	2.7	1.36	97.8
2-53	L234S/L235V/G236R	2.5	1.22	98.1
2-54	L234T/L235A/G236R	1.6	0.84	98.1
2-55	L234T/L235D/G236R	1.7	0.81	97.7
2-56	L234T/L235H/G236R	2.3	0.86	nd
2-57	L234T/L235I/G236R	2.1	0.82	nd
2-58	L234T/L235K/G236R	2.1	0.82	nd
2-59	L234T/G236R	3.0	1.28	nd
2-60	L234T/L235Q/G236R	2.6	1.31	98.2
2-61	L234T/L235R/G236R	2.9	1.50	97.9
2-62	L234T/L235S/G236R	2.6	1.29	97.8
2-63	L234T/L235T/G236R	2.7	1.35	97.7
2-64	L234T/L235V/G236R	2.0	0.80	97.7
2-65	L234A/L235A	1.7	0.76	97.8
2-66	L234A/L235A/P329G (LALAPG)	2.6	1.04	97.7
2-67	N297Q (aglycosyl)	2.4	1.00	97.9
2-68	G236R/L328R	2.5	1.04	98.1
2-69	L234A/G237A	3.0	1.35	97.5
2-71	L234A/L235E	3.4	1.58	98.0
2-72	L235V/F243L/R292P/Y300L/P396L	2.6	1.13	97.5
2-73	D265A/P329A	2.4	1.05	nd
2-74	L234A/L235A/K322A	4.1	1.74	nd
2-75	L234F/L235E/P331S	2.6	1.42	nd
2-76	L234F/L235Q/K322Q	2.4	1.03	nd
2-77	L234A/L235A/G237A/P238S/H268A/A330S/P331S	3.0	1.10	nd
2-78	E233P/L234V/L235A/G236Δ/A327G/A330S/P331S	2.5	1.01	nd
2-79	L235A/G236R	2.6	1.11	nd
2-80	L235S/G236R	2.8	1.42	nd
2-81	G236R	2.7	1.10	nd

nd = not done
