# Supplementary material for: Fc-engineered antibodies with immune effector functions completely abolished
Source: PLoS One. 2021 Dec 21;16(12):e0260954. doi: 10.1371/journal.pone.0260954 (PMC8691596; doi:10.1371/journal.pone.0260954)
Supplement: S6 Table — (PDF) [file pone.0260954.s007.pdf]

S7 Table: Yield and purity of variant antibodies produced from CHO cells

Sample	Amino acid alterations and sample description	Yield (mg/L)	Yield (relative to wt)	Purity by SEC-HPLC (%)
5-1	wild type	774.5	1.00	91.1
5-52	L234S/L235T/G236R	963.7	1.24	90.2
5-65	L234A/L235A (LALA)	897.9	1.16	88.7
