# Supplementary material for: Fc-engineered antibodies with immune effector functions completely abolished
Source: PLoS One. 2021 Dec 21;16(12):e0260954. doi: 10.1371/journal.pone.0260954 (PMC8691596; doi:10.1371/journal.pone.0260954)
Supplement: S7 Table — (PDF) [file pone.0260954.s008.pdf]

S8 Table: Preliminary screen of variant antibodies binding to human FcγRI.

Sample	Amino acid alterations	Binding response (RU)
Ab00126-10.0	wild type	391.7
Ab00126-10.16	L234A/L235A (LALA)	58.7
Ab00126-10.2	N297Q	47.0
Ab00126-10.3	L234Q/L235S	8.2
Ab00126-10.58	D265A	167.5
Ab00126-10.75	L234A/L235A/P329G (LALAPG)	2.3
Ab00126-10.77	L234Q/L235S/G236R	-0.2
Ab00126-10.79	L234A/L235A/K322A	52.2
Ab00126-10.80	L234F/L235E/P331S	37.5
Ab00126-10.81	L234F/L235Q/K322Q	72.8
Ab00126-c578	L234Q/L235S/G236K	2.2
Ab00126-c580	L234Q/L235S/G236E	0.5
Ab00126-c583	L234Q/L235S/G236K/L328P/A330P	0.8
Ab00126-c584	G236R/L328R	0.7
Ab00126-c585	L234A/L235A/G237A/P238S/H268A/A330S/P331S	11.4
Ab00126-c586	E233P/L234V/L235A/G236del/A327G/A330S/P331S	0.9

Samples of purified antibodies were injected over immobilised FcγRI for 1 min at 30 µL/min and allowed to disassociate for 3 min. The mean SPR signal (relative to the reference cell) was measured approximately 5 seconds before the end of injection (response). The blank (buffer) response was subtracted to give a corrected binding response for each test sample. This preliminary experiment was carried out using a Biacore 3000 which is less sensitive than the Biacore T200 used in the rest of the study. Furthermore, the test samples were not replicated.
